# Mapping nano-scale mechanical heterogeneity of primary plant cell walls

**DOI:** 10.1093/jxb/erw117

**Published:** 2016-03-17

**Authors:** Gleb E. Yakubov, Mauricio R. Bonilla, Huaying Chen, Monika S. Doblin, Antony Bacic, Michael J. Gidley, Jason R. Stokes

**Affiliations:** ^1^Australian Research Council Centre of Excellence in Plant Cell Walls; ^2^School of Chemical Engineering, The University of Queensland, Queensland, Australia; ^3^Australian Institute for Bioengineering and Nanotechnology, The University of Queensland, Queensland, Australia; ^4^School of BioSciences, The University of Melbourne, Parkville, VIC 3010, Australia; ^5^Centre for Nutrition and Food Sciences, Queensland Alliance for Agriculture and Food Innovation, The University of Queensland, Queensland, Australia

**Keywords:** Atomic force microscopy, cell mechanics, *Lolium*, *multiflorum*, nanoindentation, primary cell walls, suspended culture.

## Abstract

Nanoindentation experiments are performed using an atomic force microscope (AFM) to quantify the spatial distribution of mechanical properties of plant cell walls at nanometre length scales. At any specific location on the cell wall, a complex (non-linear) force–indentation response occurs that can be deconvoluted using a unique multiregime analysis (MRA). This allows an unambiguous evaluation of the local transverse elastic modulus of the wall. Nanomechanical measurements on suspension-cultured cells (SCCs), derived from Italian ryegrass (*Lolium multiflorum*) starchy endosperm, show three characteristic modes of deformation and a spatial distribution of elastic moduli across the surface. ‘Soft’ and ‘hard’ domains are found across length scales between 0.1 µm and 3 µm, which is well above a typical pore size of the polysaccharide mesh. The generality and wider applicability of this mechanical heterogeneity is verified through *in planta* characterization on leaf epidermal cells of *Arabidopsis thaliana* and *L. multiflorum*. The outcomes of this research provide a basis for uncovering and quantifying the relationships between local wall composition, architecture, cell growth, and/or morphogenesis.

## Highlight

Micromechanical maps on three plant systems universally reveal ‘soft’ and ‘hard’ domains on the cell wall surface; the observed micrometre-level spatial heterogeneity may be significant for cell growth and morphogenesis.

## Introduction

Measuring mechanical properties of cell walls presents a significant but important challenge. One of the key factors contributing to the complexity of plant cell walls is the non-uniform spatial distribution of mechanical properties such as the elastic modulus. This effect has recently been demonstrated at the tissue level for shoot apical meristems (SAMs) of *Arabidopsis thaliana*. Kierzkowski *et al.* (2012) found that hypo-osmotic treatment of the meristems causes 45–80% surface expansion of peripheral cells but only 25% for cells in the central area. These results are supported by direct nano-mechanical measurements on plasmolysed SAMs using atomic force microscopy (AFM), where the elastic modulus of cells in the apex is measured to be 5±2MPa compared with only 1.5±0.7MPa at the periphery (Milani *et al.*, 2011). The significance of such local wall mechanical heterogeneities has become increasingly relevant in a number of biological processes and was again highlighted upon discovering that the auxin transporter PIN1 responds to mechanical cues, indicating that the mechanical map of the meristem may co-ordinate polarity (Braybrook and Peaucelle, 2013).

Mechanical properties of walls are determined by the composition and organization of both the macromolecular components of the wall and the water content. These components provide structural support as well as a signalling function via a feedback mechanism that informs the interior biochemical machinery of the cell of its mechanical environment, as well as placing constraints on possible realizations of plant cell wall microstructure. The latter stems from the fact that not all microstructures that can be assembled for a given cell wall composition provide an adequate mechanical barrier that (i) counter-balances the turgor pressure; and (ii) has tuneable properties that enable cell growth and deformation. Little, however, is known about mechanical heterogeneity within walls at the cellular level.

We hypothesize that mechanical heterogeneity and the resultant local gradients of mechanical stress are important factors driving the assembly of polysaccharides at the cell wall level. Such local mechanical stresses acting in concert with intermolecular interactions such as hydrogen bonding provide a physical mechanism of formation of different types of microstructures with a wide range of mechanical properties. During their development, cells may ‘utilize’ this mechanism to adopt a certain mechanical phenotype, ‘stiff’ or ‘soft’, which is essential for the formation of growth zones and morphogenic specialization.

While there are reports suggesting mechanical heterogeneities at the level of a single cell, the magnitude of the variations remains uncharacterized. Using AFM indentation on *Arabidopsis* suspension-cultured cells (SCCs), Rodotić *et al.* (2012) observed ‘stiffness’ to range from ~20 kPa to 800 kPa. Although nano-scale mechanical heterogeneities have not been widely reported for higher plants, they are seen in yeast cells in the form of raft-like structures; the microstructure of the chitin wall is readily revealed using AFM imaging of the cell surface (Touhami *et al.*, 2003). The structural basis for mechanical heterogeneities is suggested to be associated with the microstructural arrangement of cellulose fibrils in the wall, including their density and degree of association/aggregation. Recently, the presence of cellulose fibril bundles that inform such structural heterogeneity was documented in onion (*Allium cepa*) epidermal cells (Kafle *et al.*, 2014; Zhang *et al.*, 2014) and maize (*Zea mays*) parenchyma (Ding *et al.*, 2014). These bundles, in addition to the preferred in-plane orientation of cellulose fibrils in the wall, are responsible for anisotropic mechanical properties; that is, the mechanical response to applied deformation in the longitudinal direction (along the length of fibrils) is different from that in the transverse direction (perpendicular to the fibrils’ preferred orientation) (Cosgrove, 2000, 2005; Cosgrove and Jarvis, 2012; Park and Cosgrove, 2012; Ding *et al.*, 2014; Kafle *et al.*, 2014; Zhang *et al.*, 2014). Differences in stiffness may also arise from the heterogeneous composition of the wall, which is extensively documented (McCann *et al.*, 1997; Burton *et al.*, 2010; Lee *et al.*, 2011, 2013), with nearly all constituent cell wall polymers displaying at least some level of chemical heterogeneity. We do not yet have the measurement capability to provide direct links between cell wall composition and local micromechanical properties, but a step towards achieving this goal is to provide a robust methodology for mapping nano-scale mechanical properties.

Currently, the effect of microstructure-based heterogeneities on the overall mechanical properties of tissues and plant organs is not well understood. The models applicable for direct experimental analysis are generally based on continuum mechanics (Gibson, 2005; Boudaoud, 2010; Dyson *et al.*, 2012) and do not incorporate consideration of the underlying microstructure. Microstructure is a term used to describe the appearance or arrangement of structural components (or phases) in a material across a broad range of length scales (from nanometres to centimetres). Although continuum models shed some light on static mechanical properties (Huang *et al.*, 2012), they are inadequate for developing the structure–property–function relationships that are necessary to describe the complex dynamic and transient mechanical properties of plant cell walls, plant cells, and tissues (Altartouri and Geitmann, 2015). Recently, Milani *et al.* (2014) showed that the expression patterns of some genes correlates with the elasticity of the cell walls. Observations of such correlations provide key evidence of a connection between the mechanics of the wall and its biosynthesis.

In this study, we examine the mechanical properties of plant cell walls using SCCs derived from Italian ryegrass (*Lolium multiflorum*) starchy endosperm. The use of *Lolium* SCCs enables us to probe mechanical heterogeneity in a commelinoid monocot, which, in contrast to eudicots, is rich in mixed-linkage glucan (MLG) and heteroxylans (HXs), and with relatively low levels of cellulose, xyloglucan, and pectin (Table 1). We use novel microfabricated microwell arrays to entrap cells physically without the need for clamps, sticky tape, or adhesive layers that can disturb plant material and produce artefacts associated with adhesion and uncontrolled deformation. A detailed characterization of micromechanical properties using AFM nanoindentation and our advanced multiregime analysis (MRA) routine (Bonilla *et al.*, 2015) reveals heterogeneous distribution of elastic moduli on the outer wall surface of *Lolium* SCCs, including ‘soft’ and ‘hard’ domains. We also quantify micromechanical heterogeneity *in planta* using leaf epidermal cells of *Arabidopsis thaliana* and *L. multiflorum* seedlings as a representative dicot and commelinoid monocot, respectively. The results suggest that the domain structure of mechanical heterogeneity at the micrometre level is an inherent property of plant cells and tissues, and may have significant repercussions for our understanding of cell growth and morphogenesis.

**Table 1.** Cell wall composition in molar percentage of plant systems studied using nanoindentation

	*Lolium* SCC^*a*^ (Smith and Stone, 1973*a*)	*Lolium* leaf epidermis^*b*^ (Chesson *et al.*, 1985)	Arabidopsis leaf^*c*^ (Pettolino *et al.*, 2012)
Cellulose	15.7	38.8	29.7
Xyloglucan	–	10.9	10.5
Mixed-linkage glucan	47.6	6.3	–
Pectin HG	} 4.7	} ~14	25.5
Pectin RG I			9.2
Xylan (including arabinoxylan and glucurono-arabinoxylan)	30.0	26.7	6.4
Mannan	–	+ (Tr)	3.2
Arabinogalactans (AGI and AGII)	1	+ (Tr)	6
Arabinans	–	~1	2.3
Others	~5	3.3 (including galactans)	7.2

Notations used: (~) approximate figures, (–) not detectable, +(Tr) trace amounts.

^*a*^ Cell walls isolated from *L. multiflorum* endosperm grown in liquid suspension culture.

^*b*^ Intact primary epidermis cell walls prepared from early cut leafs of *L. multiflorum.*

^*c*^ De-starched *A. thaliana* leaf cell walls, alcohol-insoluble residue preparation.

## Materials and methods

### Plant materials

#### *Lolium multiflorum* SCCs:

The *Lolium* SCCs were derived from the starchy endosperm of *L. multiflorum* grains 9–10 d post-anthesis (Smith and Stone, 1973*b*). Briefly, endosperms were placed onto solid modified White’s medium (Smith and Stone, 1973*b*) for 7 d and the resulting calli were placed into liquid medium for bulking at 27 °C in the dark in a shaking incubator at 130rpm. After establishment, the cell culture was grown in 250ml Erlenmeyer flasks containing 150ml of modified White’s medium (Smith and Stone, 1973*b*); ionic strength 0.435mol l^–1^, osmotic pressure 1.17MPa. The cultures were maintained in the dark at 27 °C with constant shaking at 130rpm. Subculturing was conducted every 10 d by weighing 30g (fresh weight) of cells and transferring the cells to 150ml of fresh medium. The polysaccharide composition of the primary cell walls of these cells was determined to be 30–50% (1,3;1,4)-β-glucan (MLG), 20–30% HX, and 5–15% cellulose.

#### *Arabidopsis thaliana* and *Lolium multiflorum* plant growth conditions:

*Arabidopsis thaliana* seeds (Columbia-0 ecotype) were surface sterilized with 70% (v/v) ethanol and 0.01% (v/v) Tween-20 for 5min, rinsed in absolute ethanol, air-dried, and individual seeds plated on half-strength Murashige and Skoog (MS) medium (Sigma) with 2% (w/v) sucrose and 0.8% (w/v) agarose (Sigma) in Nunclon Petri dishes (35×10mm, Thermo Scientific). Plates were incubated at 4 °C for 3 d in the dark then grown for 3 weeks in a growth chamber (120 µmol m^−2^ s^−1^) under a 16h day (20 ºC)/8h night (17 ºC) regime. *Lolium multiflorum* seeds were imbibed in water overnight then placed on filter paper (Whatman) in a Nunclon Petri dish and grown for 7 d in natural light (12h light, 12h dark, 22 ºC).

### Cell preparations

#### Cell preparation for AFM force curve spectroscopy (FCS) and confocal laser scanning microscopy (CLSM):

Prior to conducting analytical measurements, the *Lolium* SCCs were sieved using steel mesh sieves (ISO 3310 Test Sieves, Essa, Australia) to isolate small cell clusters and individual cells. First, a steel sieve with 300 µm mesh was used; the filtrate was then passed through a 90 µm mesh sieve. Two volumes of culture medium were used for sieving 1vol. of cells. To ensure maximum longevity of the cells, the sieving procedure was conducted every time before running AFM or CLSM measurements. Measurements were conducted within 2h of sieving.

#### Cell preparation for AFM imaging of untreated walls:

To image the surface of the cell walls, the *L. multiflorum* cells were washed with a 10× volume of White’s medium and then the medium was exchanged to de-ionized water. A copious amount of water (24 ºC) was used to remove all loosely bound components of the wall. After washing, the cell suspension was frozen overnight at –18 ºC. Before milling, samples were pre-cooled for 5min in liquid nitrogen. Cryo-milling was done in the Freezer/Mill 6850 SPEX (Metuchen, NJ, USA) for two cycles with 2min of cooling time in between the cycles; each milling cycle was performed at 10 strokes s^–1^ for 5min. The thawed suspensions of the cell wall fragments were sieved through a 90 µm mesh sieve, and the filtrate was collected. Then the filtrate was passed through a 40 µm nylon mesh cell strainer (Falcon™ Cell Strainer, Fisher Scientific), and the retentate was washed with copious amounts of water. After washing, the wet cake of the cell wall fragments with rough sizes between 40µm and 90 µm was re-suspended in 0.01% sodium azide solution in de-ionized water. The samples were kept in the fridge and used for AFM microscopy analysis within 3 d.

#### Treatment of the cells with the Updegraff reagent:

To elucidate the underlying microstructure of the cellulose network, *L. multiflorum* cells were treated using Updegraff reagent by the procedure outlined in Updegraff (1969). The reagent was prepared using 30ml of de-ionized water mixed with 120ml of glacial acetic acid and 15ml of concentrated nitric acid (both from Sigma). A suspension of cells was washed with a 10× volume of White’s medium and then the medium was exchanged to de-ionized water. Cells were then separated on a 40 µm nylon mesh cell strainer (Falcon™ Cell Strainer, Fisher Scientific) and drained under vacuum to form a moist cake. The Updegraff reagent was slowly added to the pellet; the final ratio of the reagent was 100mg mg^–1^ of cell cake. The suspension was then placed on a boiling water bath for 60min. Upon cooling, the cells were centrifuged and Updegraff reagent was replaced with water until pH 7 was reached. The samples were kept in the fridge and used for AFM analysis within 3 d.

### Microscopic characterization of *Lolium* SCCs

#### Microscopic characterization of *Lolium* SCCs using CSLM:

*Lolium* SCCs were assessed by CLSM (Zeiss LSM 710, Germany) with a three-dye staining procedure. Calcofluor white staining solution (1g l^–1^ in de-ionized H_2_O) with pre-added Evans blue (0.5g l^–1^; Sigma Aldrich) was used in 1:10 dilution to visualize glucans of the cell wall. Nile red (Sigma Aldrich) was dissolved in absolute ethanol at a concentration of 200 µg ml^–1^ to form a 100× stock solution, and was used to visualize lipid inclusions and membranes. Fluorescein diacetate (FDA) (Sigma Aldrich) was dissolved in acetone at a concentration of 2mg ml^–1^ to form a 100× stock solution, and was used to visualize/assess viable protoplasts. All dyes were used without further purification. If the fraction of viable cells after sieving was >80%, the sample was accepted for further analysis.

Additionally we performed staining using Aniline Blue Fluorochrome (Biosupplies Australia Pty. Ltd, Cat. No. 100-1) and Decolourized Aniline Blue (Mori *et al.*, 2006) for callose, but this showed no appreciable fluorescence, indicating low levels of callose in *L. multiflorum* SCC walls.

#### Microscopic characterization of *Lolium* SCCs using transmission electron microscopy (TEM):

To prepare *L. multiflorum* SCCs for TEM, small drops of liquid suspension containing cells were placed in freezer hats and high pressure frozen in a Leica PACT_2_ and then freeze substituted in a Leica AFS_2_ with a loading robot and polymerized in HM20 resin using UV. Once the resin blocks containing the *L. multiflorum* SCCs were polymerized, ultrathin sections (~80nm) were cut on a Reichert Ultracut E microtome and collected on 100 mesh copper grids. The grids containing the sections were post-stained with uranyl acetate and lead citrate. Sections were then ready for viewing on a Philips CM120 transmission electron microscope (Wilson and Bacic, 2012).

#### Microscopic characterization of *Lolium* SCCs using AFM imaging:

The AFM imaging was conducted in air in intermittent contact mode using Cypher S (Asylum Research, Santa Barbara, CA, USA). A drop of the dilute suspension of either milled cell fragments or Updegraff reagent-treated cells was placed on freshly cleaved mica and dried in air; this enabled cell fragments to adhere to the substrate. Then the samples were rinsed with water to remove all loosely adhered particles, and blow-dried with nitrogen.

### AFM force curve spectroscopy

#### FCS on *Lolium* SCCs:

AFM measurements were performed using a Nanowizard II (JPK Instruments, Berlin, Germany) mounted on an inverted fluorescent optical microscope. The Nanowizard II machine was equipped with the CellHesion module that enabled extension of the cantilever movement in the *Z* direction up to 100 µm. All measurements were performed in the closed loop mode. Indentation curves were recorded using driving speeds ranging from 200nm s^–1^ to 1000nm s^–1^ depending on the cantilever stiffness to minimize the impact of hydrodynamic drag that at all times was <5 Å in the deflection equivalent (Vinogradova *et al.*, 2001).

The probes used were pre-manufactured AFM tips; PNP-TR Si3N4 (*R* <10nm) (NanoWorld AG, Germany), DNP Si3N4 (*R* ~20nm) (Bruker AFM Probes, CA, USA), and Mikromasch NSC/CSC Si tips (*R* <10nm) (NanoWorld AG). Immediately before use, probes were cleaned in oxygen plasma for 5min and then were mounted and immersed in the experimental cuvette with buffer. The spring constant (*k*) of the sensors ranged from 0.05N m^–1^ to 1.0N m^–1^, and was determined using the Asylum Research GetReal™ routine that utilizes a combination of the thermal noise and the Sader methods (Higgins *et al.*, 2006).

A poly(dimethylsilaxone) (PDMS) microwell substrate measuring 16×23×1mm was designed to retain the individual cells *in situ* during the AFM indentation. The array contains 21 000 microwells, the diameter of which ranges from 55 µm to 75 µm in 5 µm steps to accommodate variability in cell size. The microwell array was fabricated by a standard soft lithography technique outlined in Bonilla *et al.* (2015) and Chen *et al.* (2011). The Petri dish with a microwell array was also equipped with in- and outflow tubing connected to a syringe pump. The system allows solvent exchange and perfusion of hypertonic solutions.

**Fig. 1.**
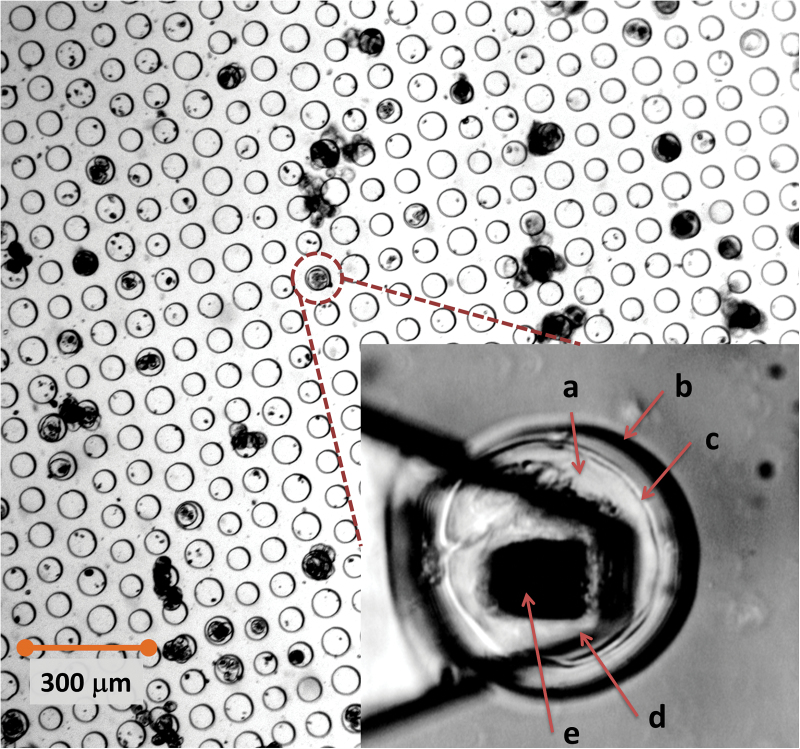
A bright-field image of a microwell array showing the distribution of cells and cell/clusters that sedimented into the well. The chance occurrence of a single cell sinking into the microwell of the matching size was utilized for AFM nanoindentation measurements. In the inset, a dual illumination (bright-field and reflected light) optical micrograph of a *L. multiflorum* SCC (a) confined within a PDMS microwell (b). The cell wall (c) is visible as a shell surrounding the cell. An AFM cantilever (d) is positioned above the cell so that the tip (e) is positioned approximately above the apex of the cell. (This figure is available in colour at *JXB* online.)

In a typical experiment, the microwell substrates were positioned in the instrument and a 1ml aliquot of filtered *Lolium* SCCs was added. After ~60s, the substrate was examined using a low magnification objective (×4/×10) to identify microwells with trapped cells of matching size as illustrated in Fig. 1. The cantilever was then positioned in close proximity to the cell, and a set of force–indentation curves (FICs) were then recorded over the PDMS substrate. This was done to cross-check the cantilever spring constant in addition to the standard calibration performed using the thermal noise method (Higgins *et al.*, 2006). It was also important to control the position of the AFM tip over the cell’s apex to ensure that the indentation is normal to the cell surface (Dufrene *et al.*, 2001). The optical image was also a way to control for any motion/rotation of the cell inside the well. The FICs were only recorded on the cells that were stable and seated firmly within the microwell. To avoid cell damage and minimize plastic deformation due to prolonged contact, the maximum indentations were limited to 500nm, and maximal forces to 500 nN, which is considered sufficient for plant cells (Fernandes *et al.*, 2012). Measurements were done in modified White’s medium filtered twice through a 0.2 µm pore size membrane filter (MillexGS MCE, Millipore, Ireland). After adding the buffer, the system was thermostated for 10–30min to ensure minimal cantilever drift. Thereafter measurements were taken on the cells. All measurements were done at 27 ºC.

For experiments with modulation of the osmotic pressure, a mannitol–trehalose solution was used to induce plasmolysis of the cells. Mannitol (Sigma Aldrich, 0.779M) and trehalose (LabChem, 1.191M) were dissolved in de-ionized water to prepare a 6.6MPa osmotic pressure solution. This solution was added using a syringe pump to elevate the osmotic pressure up to 3.5MPa. Injection speed was 50 µl min^–1^. The injection was carried out by a stepwise addition of 200 µl aliquots of osmolyte solution.

#### FCS on *A. thaliana* plants and *L. multiflorum* seedlings:

AFM measurements on plants were performed in air using a MFP-3D-BIO (Asylum Research). A Petri dish containing either Arabidopsis or *Lolium* plantlets was attached to the bottom of the stationary part of a microscope stage (i.e. underneath the *XY* AFM scanner). A leaf was then attached to the glass substrate using double-sided adhesive tape (3M™ VHB™ Tape 4920, 3M, St. Paul, MN, USA). Due to the high elastic modulus of epithelial cells, stiff cantilevers with spring constants 1 ≤*k* ≤10N m^–1^ were used [NSC 35/36 Si tips (*R* <10nm), Mikromasch, NanoWorld AG, Germany and FMG01 Si tips (*R* <10nm), NT-MDT, Zelenograd, Russia]. The force–volume two-dimensional 16×16 and 32×32 point maps were recorded with spatial density ranging from ~94nm up to ~313nm. The use of stiff tips was also required for mitigating the adhesive effect of the cuticle, which we found to be of the order of 30–50nm, which is in agreement with previous data (Kurdyukov *et al.*, 2006; Kosma *et al.*, 2009; Dominguez *et al.*, 2011). During indentation measurements, the maximum load was kept between 100 nN and 1000 nN to avoid cell puncture. The force–volume map areas were always taken around the apex of the cell to minimize the surface curvature bias (Braybrook and Peaucelle, 2013).

#### FCS data processing:

Raw force versus distance curves were recorded as a function of the voltage output from the position-sensitive device versus the calibrated *z*-position of the piezotranslator. The output voltage of the position-sensitive device was converted into a deflection in metres by calculating the slope of the constant compliance line measured against a glass/Si wafer substrate in the same buffer/solvent used to record the FIC in cells. The force was calculated by multiplying deflection by the cantilever spring constant. The zero position was determined as the cross-section point of the baseline and the tangent line corresponding to the onset of the indentation curve, where cantilever deflection started to deviate from the baseline. Positive values were attributed to the indentation section of the curve. The apparent separation was calculated by subtracting cantilever deflection from the *z*-position of the piezotranslator. No further assumptions were made as to whether the initial parts of the response corresponded to surface forces or actual indentation. Further analysis was performed using a dedicated custom-written MATLAB code that is described in detail elsewhere (Bonilla *et al.*, 2015).

## Results and Discussion

### Interpretation of force indentation profiles for small-scale localized cell deformations using an MRA

**Fig. 2.**
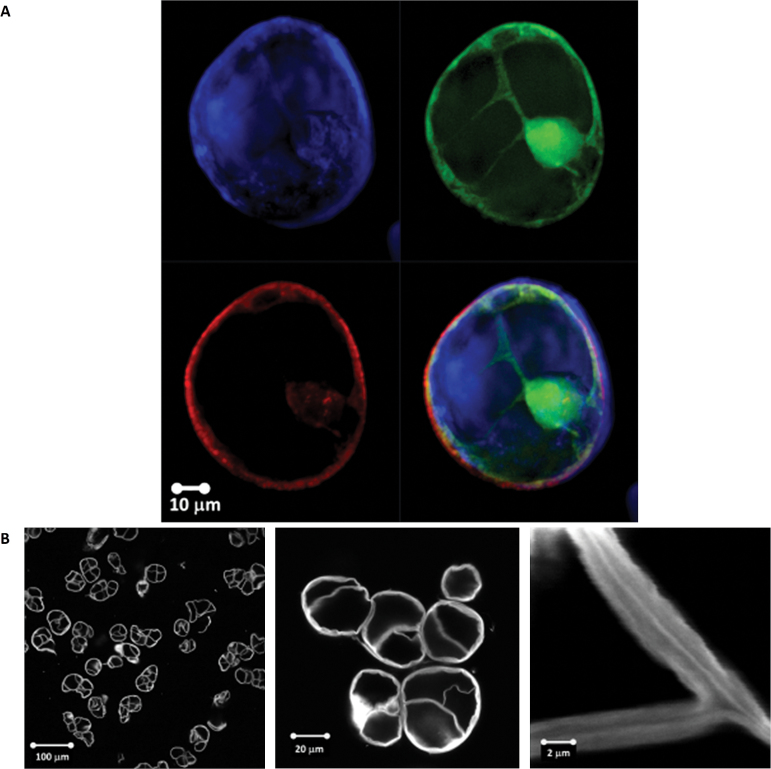
(A) A typical single cell of *L. multiflorum* SCCs. The image was taken in modified White’s medium under fully turgid conditions. The Calcofluor white staining (upper left), FDA (upper right), and Nile red (bottom left) channels show cell wall, cell cytoplasm, and lipid membrane and lipid inclusions, respectively. The overlay of all three channels is given in the bottom right panel. (B) Representative images of the *L. multiflorum* SCCs after treatment with Updegraff reagent taken at different magnifications, the cell walls were stained with Calcofluor white. (This figure is available in colour at *JXB* online.)

In Fig. 2A (and Supplementary Fig. S1 at *JXB* online), a typical individual cell of *L. multiflorum* SCCs is shown under fully turgid conditions. The size of the cells ranges from 20 µm to 100 µm. The thickness of the cell wall, as indicated by the Calcofluor white staining, is between 0.5 µm and 2 µm; this result is further verified by TEM imaging (Supplementary Fig. S2). In Fig. 2B, the representative images of cell ‘ghosts’ after treatment with Updegraff reagent show that the cellulose subnetwork of the wall is continuous, and its thickness is comparable with the thickness of unmodified walls. These observations led us to a mechanical model of the SCC as a thick-walled pressurized shell.

The presence of the wall makes deformation of plant cells complex; a number of mechanical responses occur simultaneously and often with indistinguishable contributions towards the force–indentation profiles (for a conceptualized overview of mechanical parameters used in interpreting complex deformations, see Supplementary Fig. S3; Supplementary Table S1). All types of deformations can be broadly divided into two categories: reversible (or elastic) and non-reversible (or dissipative). The former correspond to a spring-like action; that is, the substrate deforms under the applied force, but reverts back to its original shape once the force is released. The latter category is when the deformation profiles during compression and decompression differ. Typical examples of non-reversible deformations are viscous, plastic, and viscoelastic deformations.

**Fig. 3.**
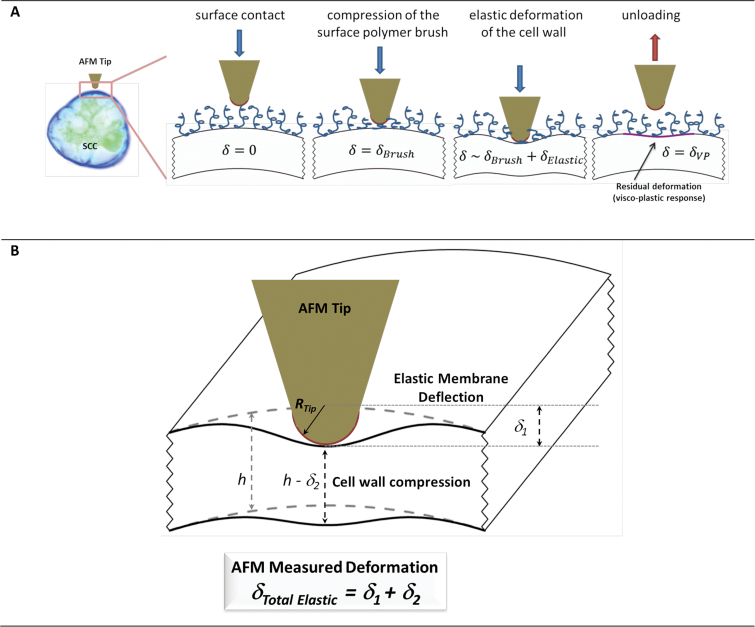
(A) A schematic representation of a postulated deformation sequence during the indentation of an SCC using a blunt AFM tip. (B) A physical model of plant cell wall deformation during the nanoindentation with an AFM tip. The AFM spring (with a constant, *k*_AFMcantilever_) is balanced by the elastic response from cell deformations that depend on the material properties of the wall; the elastic membrane spring constant (*k*_M_) and effective modulus of the wall $({\tilde{E}}_{wall}).$ The latter is a function of the Young’s elastic modulus (*E*) and the Poisson ratio (*v*). (This figure is available in colour at *JXB* online.)

The elastic (reversible) deformations in plant cell systems are non-trivial to interpret. During the indentation cycle (Fig. 3A), the cell wall deforms, as does the turgid protoplast. The total indentation in this example comprises two contributions; one is from the cell wall compression, and another from its bending, as illustrated in Fig. 3B for the case of a blunt AFM tip indenting a small segment of the wall. Although both deformations are assumed to be elastic, the complexity stems from the anisotropy of the wall (properties in transverse and longitudinal directions are different), and the presence of a turgid protoplast. These factors result in a scenario where the observed deformation profile reflects different cell wall properties. The compression component is primarily associated with the transverse Young’s modulus of the wall and its transverse Poisson ratio. The bending can be characterized using an effective membrane spring constant, which is a function of the geometric curvature of the wall, turgor pressure, the longitudinal Young’s modulus, as well as a number of other parameters such as the cell wall shear modulus. (Supplementary Table S1).

We interpret this complex mechanical response using an MRA that incorporates multiple mechanical models (Bonilla *et al.*, 2015), whereby the scenario in Fig. 3B is represented as a system of ‘springs’ or ‘mechanical resistors’ that are connected in a series (Li and Bhushan, 2002).

The MRA and the corresponding automated routine allow interpretation of multiple deformation regimes that may be convoluted within a single force–indentation test. The method also ensures that force analysis complies with assumptions and applicability limits of mechanical models.

**Fig. 4.**
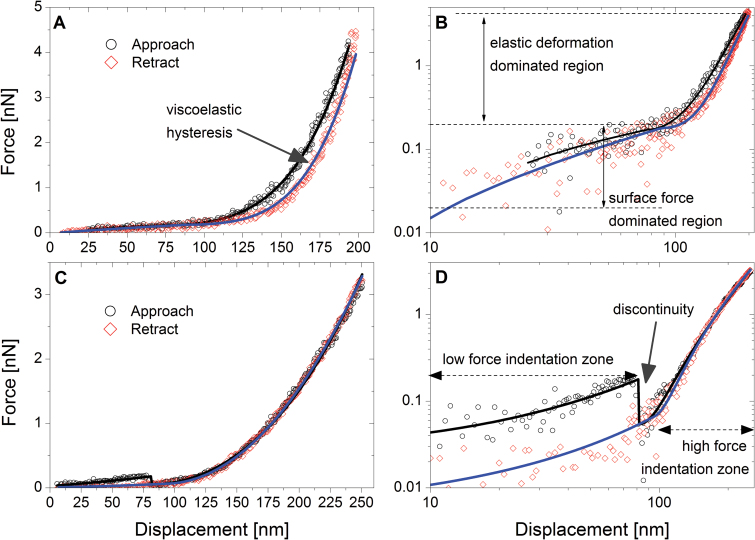
Typical force–indentation curves (FICs) for *L. multiflorum* SCCs (A and C) and their corresponding versions in log–log scale (B and D). The majority of FICs were characterized by three resistors: a surface force, linear deformation (elastic membrane), and a thin elastic film. The elastic membrane deformation occurred concomitantly with the other resistors, generating a two-regime response. In both cases, discontinuities associated with penetration of the tip into the voids of the cell wall mesh are present. (This figure is available in colour at *JXB* online.)

In Fig. 4 we present typical examples of indentation curves collected from *L. multiflorum* SCCs plotted in linear (Fig. 4A, C) and logarithmic (Fig. 4B, D ) co-ordinates. The latter enables a better visualization of low force deformations, which otherwise may be obscured by other mechanical responses that dominate the force–distance profile (Bonilla *et al.*, 2015).

A typical FIC is a sequence of compression and decompression tests (Fig. 3A). The compression is recorded first when the AFM cantilever tip indents the sample, while the decompression is recoded when a tip is retracted from the surface. The low force regime is dominated by a weak repulsive interaction which spans the indentation depth anywhere between 50nm and 500nm. Further indentation results in a high force region dominated by the mechanical deformation of both the cell and cell wall (Fig. 3B). Figure 4 also illustrates the magnitude of the hysteresis between the approach and retraction curves that is suggestive of contributions from both viscoelastic and plastic deformations. The moment the tip detaches from the surface, an adhesive interaction may manifest itself by producing a pull-off peak. Typically little or no adhesion is observed, although at certain locations on the cell surface multiple detachment peaks are recorded, consistent with stretching of surface-bound polymers (Supplementary Fig. S4). Due to the rarity (~1 in 20 curves) of such events, the detailed analysis was not performed; however, a qualitative evaluation suggests that the most likely mechanism of these interactions is through unspecific physical entrapment of loosely attached polysaccharides by the AFM tip during its exit from the wall. Another feature of the indentation profiles is that a significant number of curves have discontinuities, as shown in Fig. 4C and D. These discontinuities are likely to be associated with the penetration of the tip into the voids of the polysaccharide mesh of the wall.

In addition to the MRA of the force–indentation data, the dissipative parameters such as the area of hysteresis (proportional to the dissipated energy) and the plastic deformation have been recorded. The latter is measured as a distance between the approach and retract branches of the FIC at the point of zero force (for reference, see Supplementary Fig. S3B). These measures provide important insights into possible dissipative mechanisms within the system, and therefore have to be considered in interpretation of the results from MRA analysis.

The majority of data sets recorded for *Lolium* SCCs using an AFM tip display a behaviour characterized by a three-resistor model that describes three types of deformation outlined in Fig. 3A and B: (i) polymer steric repulsion model, *n*_1_ <1 (Balastre *et al.*, 2002; Iyer *et al.*, 2009; Bremmell *et al.*, 2013); (ii) elastic membrane model, *n*_2_ ~1 (Begley and Mackin, 2004; Vella *et al.*, 2012); and (iii) thin film elastic model, *n*_3_ ≥2 (Dimitriadis *et al.*, 2002; Gavara and Chadwick, 2012). The latter is an extension of a well-established Hertzian–Sneddon model (Hertz, 1882; Sneddon, 1965) that accounts for a finite thickness of the deformed material, as is the case with plant cell walls (see Supplementary Model S1 for details of each deformation model and the equations used).

First, the low force regime with *n* <1 is attributed to the interaction of the tip with loosely adsorbed or protruding polymers, which are evident from TEM images (Supplementary Fig. S2). Such dependency is consistent with a mechanism whereby ‘grafting’ density of polymer chains changes with separation. This can be realized either for a polydisperse brush where density increases as the tip ventures deeper into the polymer layer, and starts to probe shorter chains, or through the lateral displacement of polymer molecules originating from the conical shape of an AFM tip that effectively wedges in between the chains (Balastre *et al.*, 2002; Bremmell *et al.*, 2013; Cuellar *et al.*, 2013). The presence of such a loose polymer layer is consistent with the fact that walls of *L. multiflorum* SCCs have a relatively low content of cellulose (<15%/dry weight), but are rich in MLG and arabinoxylan (Table 1).

The second regime with 0.9 <*n* <1.4 is identified as a combination of two resistors, *n* ~1.5 and *n* ~1; note, if these occur concomitantly, an intermediate value of exponent is observed. A power law response with *n* ~1.5 is predicted for a Hertzian contact deformation exerted by a spherical punch, which is satisfied for small deformation when δ *<*2×*R*_Tip_. A linear response (*n* ~1) is predicted by theories for deflection of membranes and localized (central) deformations of spherical shells, provided that the relative deformation $\varepsilon =\frac{\delta }{R_{cell}}\ll 1$ which is easily satisfied in the small deformation AFM experiments. The values of the spring constant in the MRA model do, however, depend on the scenario that is at play (Begley and Mackin, 2004; Vella *et al.*, 2012). For small deformations of a pressurized spherical shell, *K*_M_ is estimated to be 50N m^–1^ when *P*_turgor_=3 MPa (Arnoldi *et al.*, 2000). However, the measured values of the effective membrane spring constant are found in the range between 0.01 and 2, which is consistent with the case of an unpressurized spherical shell in the bending-dominated regime (Vella *et al.*, 2012) or, alternatively, with the indentation of a flat membrane (Begley and Mackin, 2004). In the latter case, one has to assume a membrane cut-off area with radius smaller than $\sqrt{R_{cell}h},$ where *h* is cell wall thickness. We always observe this regime to precede the transverse elastic deformation of the wall, which suggests that the characteristic bending length is limited to very narrow areas on the cell surface (*l*_B_ <<*h*) (Dumais, 2007; Audoly and Boudaoud, 2008; Sultan and Boudaoud, 2008). Such a scenario is consistent with the tip indenting the areas in between polysaccharide fibres, and where the cellulose fibre mesh would act as a boundary for local deformations due to its intrinsic stiffness.

The third deformation regime observed is consistent with the indentation of the cell wall in the transverse direction, for which 1.5 <*n* <4. The lower boundary (*n*=1.5) corresponds to the Hertzian deformation exerted by a spherical indenter, which is consistent for an AFM tip at small indentations that is truncated as a cone or pyramid. For larger indentations, a conventional Sneddon’s solution for a cone predicts *n*=2. For a majority of FIC curves, however, larger values of the exponent are observed that we attribute to a non-linear deformation of the thin wall, where deformation is comparable with wall thickness (δ *┴ h*).

We assume that for *n* >4 the system is at the limit of the linear (or quasi-linear) elastic approximation; thus, these regions were excluded from the analysis.

### Mechanical mapping of individual cells of *Lolium* SCCs

**Fig. 5.**
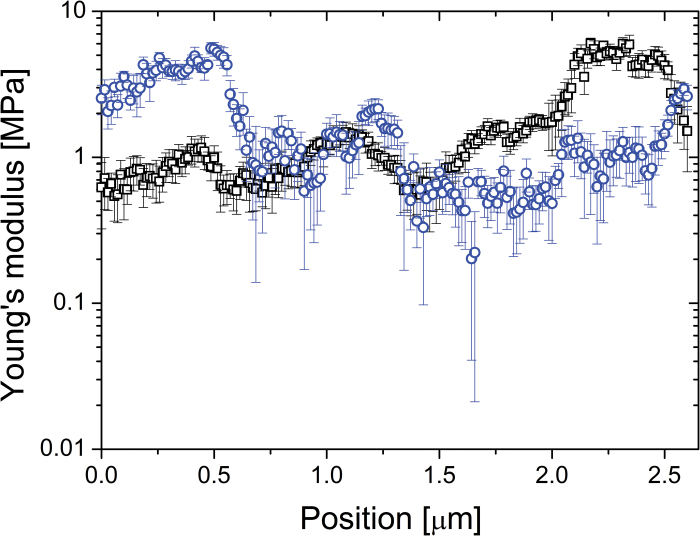
Typical traces of the effective Young’s modulus as a function of the position on the cell surface recorded on two different *Lolium* SCCs (rectangles and circles) along a 2.6 µm path. The data are taken along a straight arc over the cell’s surface (~1/20 of the cell radius). Two repetitions per point were performed; the symbols represent the average of four values (two repetitions, each being characterized by two values of the modulus calculated using approach and retract branches of the indentation curves); the bars represent deviations. The track was recorded within the time interval of 15min. (This figure is available in colour at *JXB* online.)

In order to map the mechanical properties of the *Lolium* SCC walls reliably, numerous 1D tracks are recorded with 2–3 curves per point and ~100–300 curves per track. By doing so, the distance between spatial points is less than ~30nm, which enables probing of whether the change in the modulus is a continuous function of the position or random. Figure 5 shows typical traces of the effective Young’s modulus as a function of the position on the cell surface recorded on two different cells along a 2.6 µm path. The observed variations in the elastic parameters greatly exceed the accuracy of the measurements at each point, and the measured distribution of heterogeneity is consistent with the hypothesis of micromechanical domains. As shown previously (Bonilla *et al.*, 2015), the high level of accuracy is also ensured by extracting elastic parameters from both approach and retraction parts of the indentation curve, from which the MRA routine isolates Hertzian components from other types of force response

**Fig. 6.**
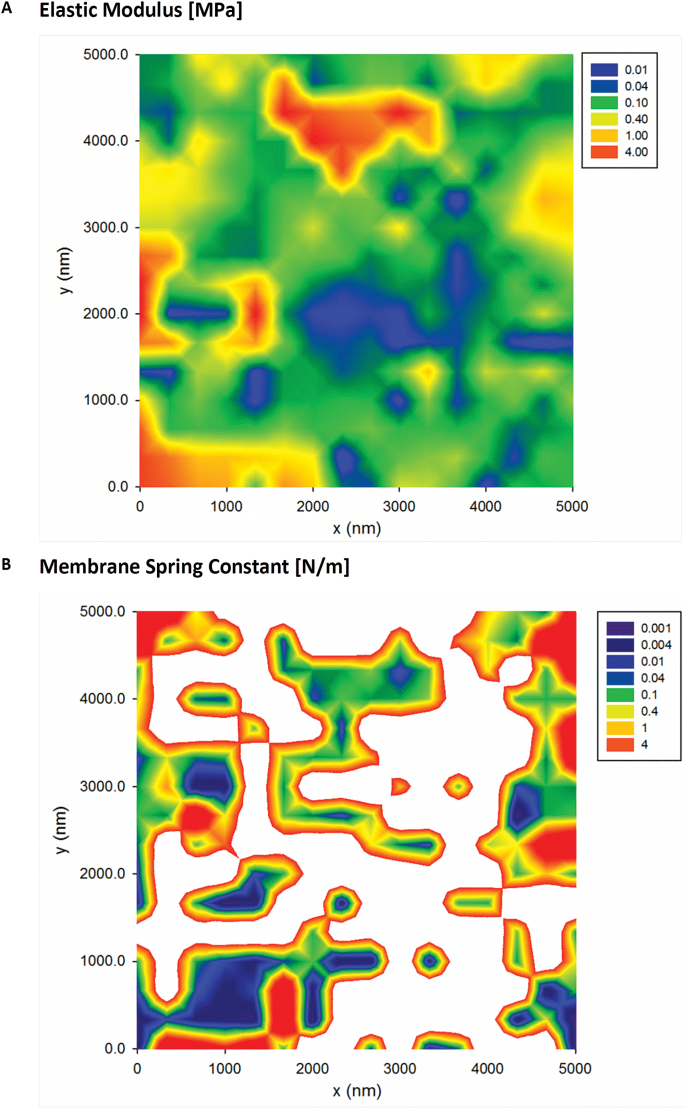
2D force–volume plots of the elastic modulus (A) and the membrane spring constant (B) of *Lolium* SCCs. All plots were recorded over a 5×5 µm area. The white areas on the spring constant map (B) correspond to the areas where determining such a type of deformation was not experimentally possible. (This figure is available in colour at *JXB* online.)

To illustrate further the presence of mechanical heterogeneities, we have collected 2D force volume plots over 5×5 µm areas. Figure 6 presents a typical map of the effective Young’s modulus and membrane spring constant. The maps allow measurement of the variation in elastic modulus (Fig. 6A) which is found to span at least three orders of magnitude, between 0.01MPa and 10MPa. The median and mean are 120 kPa and 700 kPa, respectively, which are similar to those previously reported for AFM measurements on single plant cell systems (Milani *et al.*, 2011; Radotic *et al.*, 2012). The maps of the membrane spring constant, *k*_M_, presented in Fig. 6B reveal similar variations in the magnitude and the spatial distribution. The membrane spring constant is related to the longitudinal elastic modulus of the wall in a localized area around the AFM tip, hence both maps provide complementary data characterizing mechanical heterogeneity of the anisotropic wall.

**Fig. 7.**
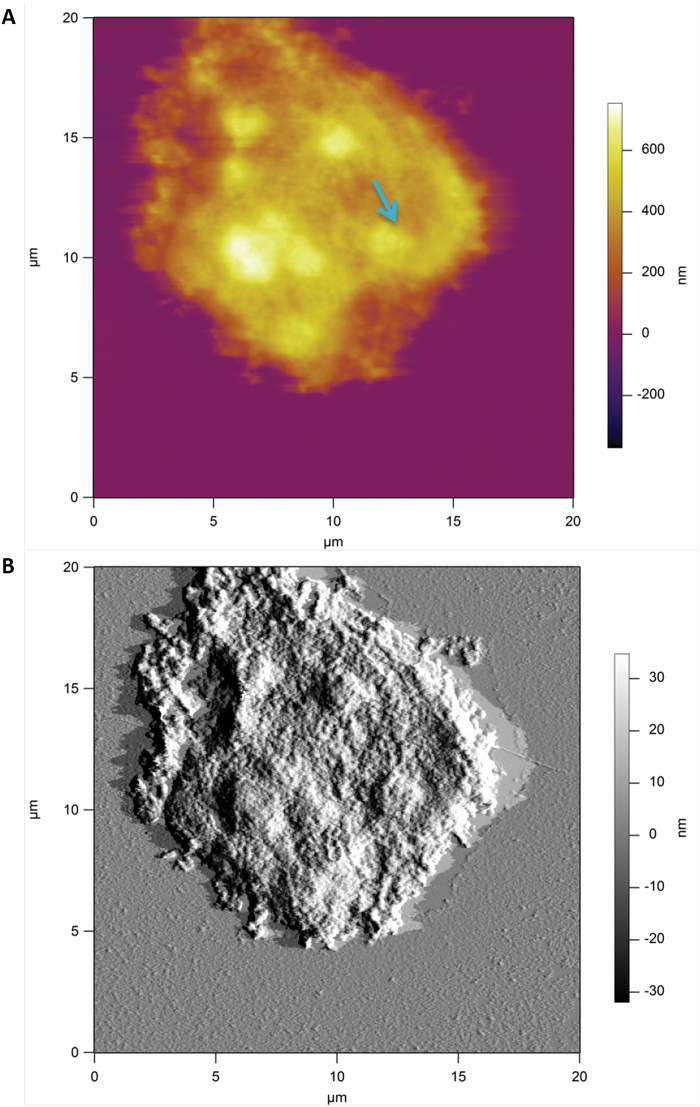
Height (A) and amplitude (B) AFM images of a wall fragment of *Lolium* SCCs obtained via cryo-milling. The sample was deposited on mica and imaged in the intermittent contact mode in air. The surface of this wall fragment is dominated by the amorphous layer of polysaccharide. Some features in the form of ‘blobs’ with above average height (indicated by an arrow) may be indicative of underlying microstructure. (This figure is available in colour at *JXB* online.)

The key finding is that the spatial distribution of heterogeneities of both the Young’s modulus and membrane spring constant has a clear domain structure, covering length scales from several hundreds of nanometres up to a few micrometres. This is because the presence of large-scale domains of mechanical heterogeneities, with the Young’s modulus varying spatially by at least two orders of magnitude, is suggestive of heterogeneity in the microstructure of the deposited cell wall polysaccharides. Figure 7 shows a typical AFM image of the surface of a cryo-milled *Lolium* SCC wall fragment washed with de-ionized water, which reveals an apparently homogenous and amorphous surface. We note that the deposition of cell wall fragments on the mica surface is random and hence some of the surfaces are representative of the outer surface of the wall and others of the inner surface of the wall, which is in contact with the plasma membrane. From imaging alone we are unable to distinguish between these surfaces. These results suggest that the surfaces of *Lolium* SCCs are probably covered by loosely associated non-cellulosic polysaccharides, masking any underlying heterogeneity.

**Fig. 8.**
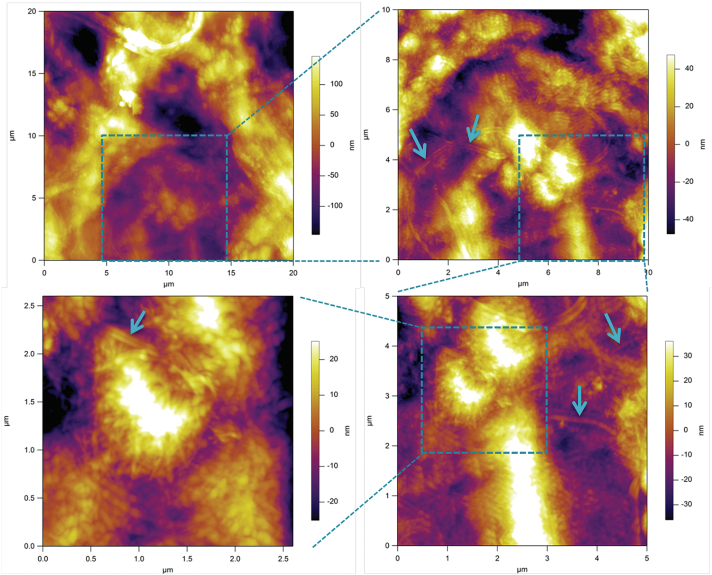
An AFM image of the surface of a *Lolium* SCC ‘ghost’ after treatment with Updegraff reagent showing the heterogeneous deposition of cellulose in the wall. Images are taken with increasing magnification, as indicated by boxed areas. Arrows show some representative fragments of acid-resistant cellulose fibrils. The longer fibrils appear as if they are connecting the tufts of tightly packed shorter fibrils. (This figure is available in colour at *JXB* online.)

To obtain more detailed representation of the underlying cellulose network, we have treated SCCs with Updegraff reagent. The results presented in Fig. 8 show a heterogeneous distribution of the acid-resistant fragments of cellulose fibrils. The key features of this microstructure are that heterogeneity covers a number of length scales, from tens of micrometres to a few hundred nanometres, as well as containing domains with varying density and orientation of cellulose fibrils. The smallest length scale of such microstructural features corresponds well to that found on the mechanical maps, as can be gauged by comparing images in Figs 8C and 6 (5×5 µm area), and Fig. 8D with a 1D track in Fig. 5 (2.6 µm).

**Fig. 9.**
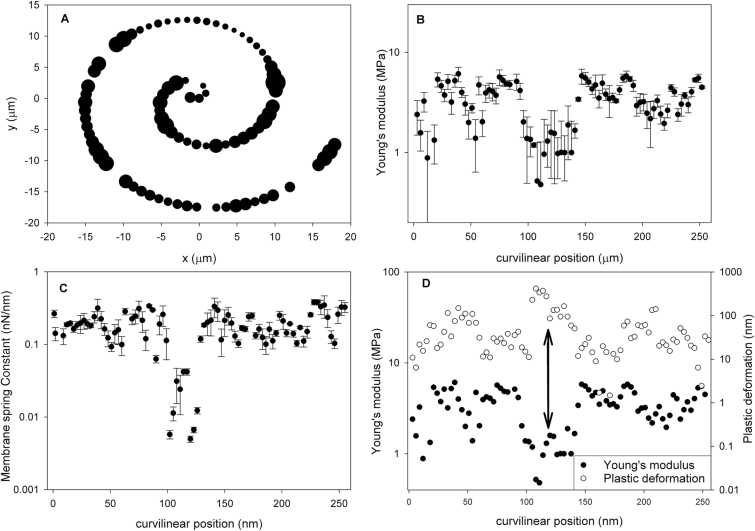
Comparison of mechanical properties evaluated for 1D tracks calculated using different models. (A) Spiral-like trajectory of indentation onto the surface of a *Lolium* SCC; the size of the symbols is proportional to the average MRA elastic modulus at the specific point. (B) Average Young’s modulus obtained along the curvilinear position; the origin represents the origin of plot (A). (C) The elastic membrane spring constant. (D) Correlation between MRA modulus and plastic deformation.

The imaging results (Fig. 8A, B) are also suggestive of the presence of even larger scale heterogeneities that may span a few tens of micrometres. In order to test this hypothesis, we performed indentation experiments to generate 1D tracks over a larger area by recording along a spiral trajectory. Figure 9 shows a typical result obtained using a fully turgid cell. The size of the circles in Fig. 9A is proportional to the average Young’s modulus obtained using MRA at a specific point, suggesting the presence of well differentiated soft and stiff areas in the wall. These are shown in more detail in Fig. 9B, where the modulus data are plotted versus the curvilinear position, with *x=0* representing the origin of Fig. 9A. One can see a clear minimum in modulus between 100 µm and 150 µm. The location of this minimum also corresponds to a minimum in the elastic membrane spring constant *k*_M_ (Fig. 9C) and a maximum in plastic deformation (Fig. 9D). We note that for the majority of samples tested, no correlation between plastic deformation and the elastic modulus is observed, which indicates that the observed correlation in a specific location is indeed indicative of an underlying physical mechanism rather than an artefact of analysis. The results clearly show the presence of distinct ‘soft’ regions of the cell wall.

The findings reported here are in a good agreement with recent results by Zhang *et al.* (2016), where reticulated deposition of cellulose fibrils as well as heterogeneous spatial distribution of soft and rigid matrix polymers (i.e. pectins and cellulose) in the walls of onion epidermis walls have been extensively documented. We note that measurements by Zhang *et al.* (2016) are performed on a recently deposited layer of the wall; that is, the images are taken of the inner layers adjacent to the plasma membrane. In that respect, such a pattern of deposition may share similarity with the outer regions of the *Lolium* SCC walls probed in our experiments. It is possible to suggest that in the walls of *Lolium* SCCs formed without restrictions of neighbouring cells, the bundled and reticulated structure of cellulose deposits may persist through to the outer layers. The heterogeneous deposition of the wall, as well as some irregular deposits in the outer surface of the wall, are also reported in the work by Digiuni *et al.* (2015), who investigated callus-derived single cells of *A. thaliana*.

It is therefore possible to conclude that observed domains of mechanical heterogeneity do correspond to the irregular and bundled deposition of cellulose and to that of the corresponding subnetwork of hemicelluloses and/or pectins.

### *In planta* mechanical mapping of leaf epidermal cells of *A. thaliana* and *L. multiflorum*

**Fig. 10.**
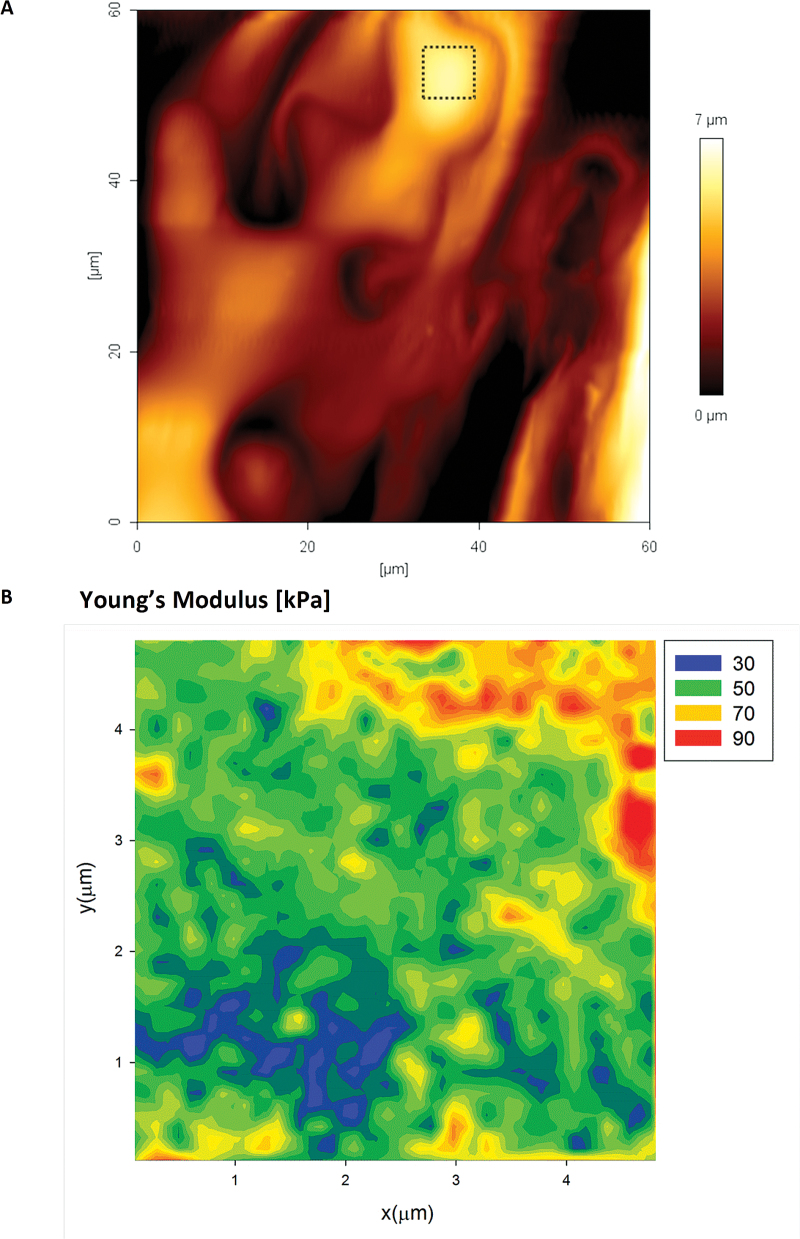
AFM height image (A) of the abaxial epidermal layer of an *Arabidopsis thaliana* leaf epidermal cell. The apex area on one of the cells (boxed area) was also tested using the 2D force–volume mapping technique. The mechanics data are well described by the Hertzian model, with maps of the Young’s modulus (B) showing strong evidence for the presence of heterogeneities. Pixel size 156nm, indenter radius 20nm. (This figure is available in colour at *JXB* online.)

In order to test the nano-scale distribution of the elastic modulus on mature cell walls within a tissue/organ, we tested abaxial and adaxial epidermal pavement cells of *A. thaliana* and *L. multiflorum* leaves. Figure 10A shows a typical AFM height image of the abaxial surface of an *A. thaliana* epidermal pavement cell. The mechanical tests were performed in the 2D force volume regime (32×32 pixels) across a 25 µm^2^ area. The map of the Young’s modulus (Fig. 10B) shows spatial variability qualitatively similar to that observed in *L. multiflorum* SCCs. The variation in the modulus for the epidermal cells (both adaxial and abaxial) is markedly smaller than for *Lolium* SCCs; we suspect this reflects how the more mature epidermal cells have a denser distribution of polysaccharides. Also since the walls have a polylamellate structure of cellulose deposition, we expect the outer lamella to bear less resemblance to the meso-scale structure of the freshly deposited lamellae (Zhang *et al.*, 2016).

**Fig. 11.**
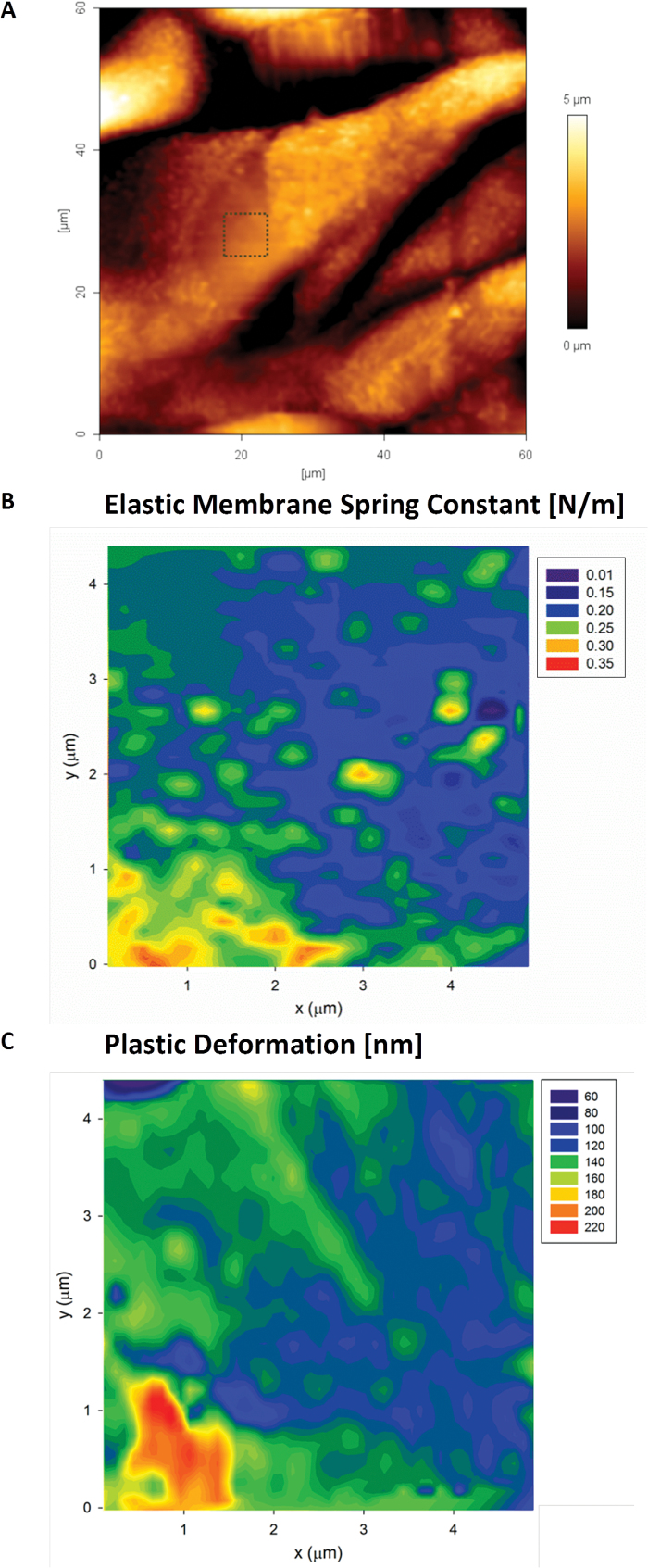
AFM height image (A) of the abaxial epidermal layer of a *L. multiflorum* leaf epidermal cell. The area on one of the cells (boxed area) was also tested using the 2D force–volume mapping technique. The mechanics data are well described by the elastic membrane bending model (B) that shows the presence of heterogeneities. The map of the plastic deformation (C) recorded over the same area showed significant correlation with the elastic spring constant. Pixel size 156nm, indenter radius 20nm. (This figure is available in colour at *JXB* online.)

The MRA analysis of 2D force–volume maps from indentation of *L. multiflorum* leaf epidermal cells (abaxial) show a marked difference compared with *A. thaliana*. Most of the data sets are well described by a single elastic membrane resistor, as shown in Fig. 11. This feature is easily detected in the histogram analysis of the power law exponents, which led us to run the MRA with membrane and Hertzian resistors; the latter in anticipation of a small portion of the data with *n* >1. Interestingly, areas of high *K*_M_ correspond well to areas of low plasticity, and vice versa. The fact that two parameters estimated through independent methods agree with each other suggests that the soft areas are not only less stiff but also display non-linear yielding behaviour characteristic of viscoplastic deformation.

We interpret the experimental findings on the three systems studied based on the cell wall composition of each cell type as summarized in Table 1. The *Lolium* leaf epidermis has the highest cellulose content, which coincides with the minimal transverse deformation of its walls, and the force response is dominated by the membrane-like deformation, namely the ‘thin-walled balloon’ model. It should also be noted that the xyloglucan content that may mediate the links between cellulose fibrils is similar in *Arabidopsis* and *Lolium* leaves, and hence one has to be careful with attributing differences in the mechanical response to the difference in the level of xyloglucan-mediated tethers or to the effect of xyloglucan on cellulose–cellulose interfibre links (Park and Cosgrove, 2012). This is further supported by the fact that *Lolium* SCCs have only a very low level of xyloglucans, if any, yet the wall is characterized by an appreciable elastic modulus in the order of megapascals, which is higher than that of *A. thaliana* leaf epidermis cells. In part, this can be attributed to the postulated layered structure of the *A. thaliana* walls, with the outer layer being softer than the inner layer (Radotic *et al.*, 2012; Digiuni *et al.*, 2015). Another possible explanation may be the lubricating effect of pectin on cellulose–cellulose interfibre junctions to effect lower resistance of the wall to deformation.

The substantial presence of MLG and pectin in *Lolium* SCCs and *A. thaliana* epidermal walls, respectively (Table 1), results in softer walls, compression of which can be measured even at small indentations. This is in contrast to the *L. multiflorum* epidermis cells that exhibit undetectable cell wall compression, with the mechanical response being dominated by the wall deflection. We note that the average values of the membrane spring constants, *k*_M_, for *Lolium* SCCs and *L. multiflorum* leaf epidermis are of the same order of magnitude, which indicates that elastic properties in the longitudinal direction (in the plane of the wall) are not too dissimilar. It is possible, therefore, to deduce that the absence of the wall compression modes in *Lolium* leaf does not stem from the fact that its wall is ‘softer’ in the longitudinal direction, and the apparent absence of wall compression is likely to be associated with the lower levels of MLGs and pectins. This conclusion is consistent with the force curves for *Lolium* SCCs and *A. thaliana* leaf epidermis cells which feature well-pronounced deformations associated with a fluid-like layer which precedes the elastic deformation of the wall, and which is consistent with a layer of gel-like polysaccharides such as MLG or pectin.

Based on the different composition of the walls in the plant systems studied, it is also possible to propose a hypothesis about the role of xylans in the mechanics of the wall, as both *Lolium* systems feature similar and rather substantial amounts of xylan (~30 molar %). One possible mechanism is via poroelastic control of water permeability that creates a condition where fluid resistance results in the elastic-like response. Such pressurization may also contribute to the non-linear wall deformation that was observed for *Lolium* SCCs, whereby the effective elastic modulus increases with indentation. The role of xylans can also be inferred from the observed values of the plastic deformation characteristic for both *Lolium* systems. Such irreversible deformation would be consistent with the viscous drainage of water-solubilized xylan during poroelastic deformation (Lopez-Sanchez *et al.*, 2014, 2015; Bonilla *et al.*, 2016). We also note that although the values of maximum indentations used in this study are of the order of a few micrometres, they are still insufficient to induce a buckling transition within the area around the indenter which also could manifest itself as an irreversible deformation (Nasto *et al.*, 2013). The buckling zone is expected for indentations of the order of $\sqrt{R_{cell}\cdot h_{wall}},$ which for a typical cell with the radius of 30 µm and wall thickness of 3 µm are of the order of 8 µm (Paulose and Nelson, 2013).

### Non-turgid cells of *Lolium* SCCs

**Fig. 12.**
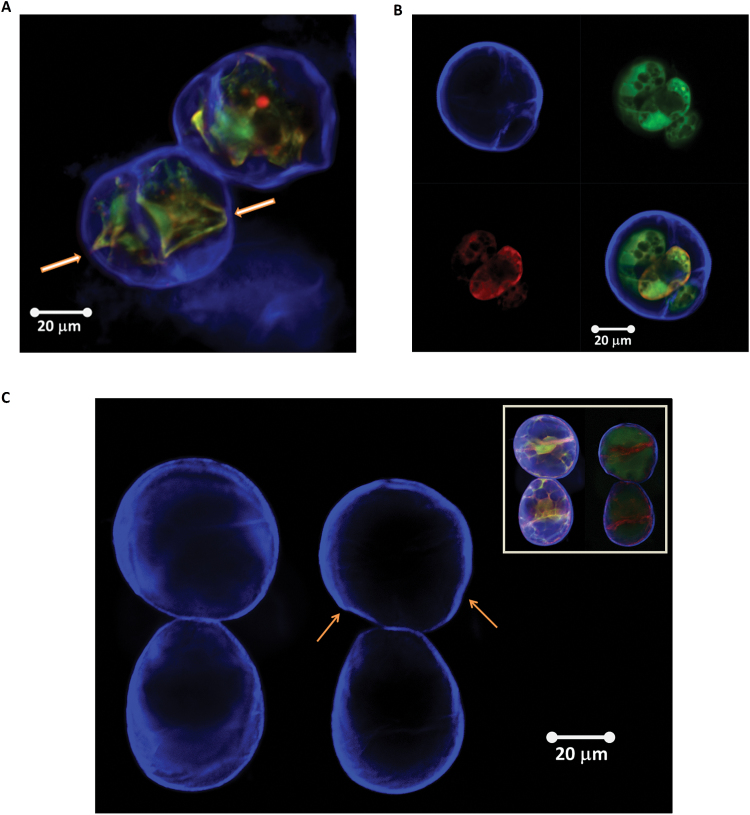
Typical examples of *Lolium* SCCs after plasmolysis (mannitol–trehalose solution) in which either partial detachment (arrows show residual attachment points) (A) or full detachment (B) of the protoplast from the cell wall was observed. In (B), staining with Calcofluor white (upper left), FDA (upper right), and Nile red (bottom left) shows cell wall, cell cytoplasm, and lipid membrane and lipid inclusions, respectively. The overlay of all three channels is given in the bottom right panel. (C) *Lolium* SCC walls before and after plasmolysis. The inset shows additional Nile red and FDA staining to visualize changes in the cytoplasm following the osmotic treatment. The arrows point to the areas of buckling and regions of irregular deformation (both in the top cell). Deformation of the bottom cell is mostly homogenous and can be used as a reference. (This figure is available in colour at *JXB* online.)

In Fig. 12A and B, representative examples of plasmolysed (non-turgid) cells are presented. One can see that plasmolysis at its extreme causes either partial (Fig. 12A) or complete detachment of the protoplast (Fig. 12B). This serves as an important validation that, at least at the highest value of the differential osmotic pressure, the plasma membrane is not in contact with the wall, and hence the mechanical properties of the wall would be close to those of an unpressurized elastic shell. We note that even under turgor, the shape of the cells often deviates from perfectly spherical (see Fig. 2A). This non-spherical shape of the majority of cells suggests that the magnitude of the turgor pressure is not sufficient to put the wall under a significant level of pre-stress. In other words, the SCCs deposit a wall that is tougher than required to counter-balance the turgor pressure. Hence results for plasmolysed SCCs should be treated with a degree of caution, as they may not fully reflect the behaviour of cells in tissues.

Figure 12C shows the same cells before and after plasmolysis. One can clearly see patterns where deformations around the circumference of the cells are non-homogeneous. Some regions (indicated by the arrows on the figure) appear to have undergone buckling transition, which provides indirect, although visually clear evidence for the presence of mechanical heterogeneities. The analysis by Paulose and Nelson (2013) shows that mechanical and geometrical heterogeneities of the shells greatly reduce the threshold of buckling, making buckled shapes less sensitive to imperfections. This predicts, in a rather counter-intuitive fashion, that introducing heterogeneity could make the buckling transition more reliable than for a uniform shell.

**Fig. 13.**
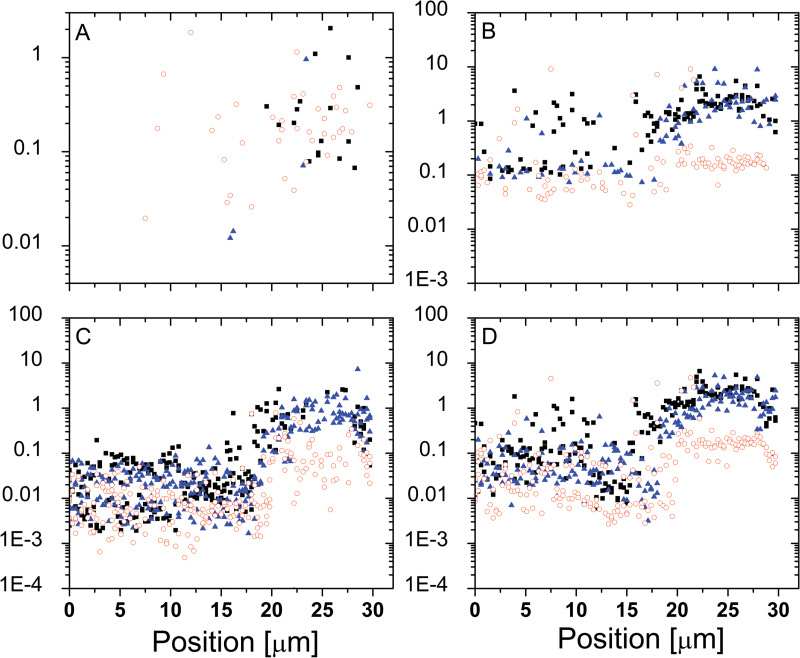
Mechanical properties recorded across a *Lolium* SCC before and after plasmolysis. The filled symbols (squares and triangles) correspond to the two consecutive tracks and illustrate the reproducibility of the measurement and analysis. The open symbols correspond to the data recorded after plasmolysis along the very same trajectory. (A) Membrane spring constant (N m^–1^). (B) Hertzian Young’s modulus (MPa). (C) Thin film elastic modulus (MPa). (D) Average elastic modulus (MPa). (This figure is available in colour at *JXB* online.)

The 1D track method was used to map the mechanical properties of *Lolium* SCCs before and after exposure to high osmotic pressure solutions. Figure 13 presents an example where selected mechanical properties are recorded along a 1D track over an ~30 µm arc on the cell surface, thus covering a distance comparable with the cell’s diameter. One can see two microscopic domains, a stiffer one (between ~20 µm and 30 µm) and a softer one (between 0 µm and ~20 µm). Upon changes in osmotic pressure, the elastic modulus of the softer domain did not exhibit much change, while the opposite is observed for the stiffer domain, where the elastic modulus dropped. These results suggest that these two locations differ in the level of pre-stress they exhibit: the higher modulus should be attributed to the areas where wall properties are influenced by strain stiffening. Upon a reduction in differential pressure and subsequent relaxation, the changes in modulus correspond to the reduction in strain stiffening. This interpretation is also supported by the fact that the non-linear elastic modulus (which effectively probes stiffening properties) shows a much more modest reduction compared with the Hertzian modulus.

The membrane spring constant showed little change in response to variation in osmotic pressure, which supports the hypothesis that this quantity is associated with the localized deformation of the domains of gel-like cell wall material confined between cellulosic scaffolds. Indeed, the bending deformations are limited by the longitudinal extensional elastic modulus of the wall (Arnoldi *et al.*, 2000; Boulbitch, 1998, 2000; Boulbitch *et al.*, 2000), which, due to the predominantly longitudinal orientation of cellulose fibrils, is expected to be much higher than the transverse one (Gutierrez *et al.*, 2009; Yi and Puri, 2012).

In comparison with other nanoindentation studies on plant cells, where no effect of osmotic pressure is found, we emphasize that these were restricted to much smaller deformations than in our current study. This includes the study of Milani *et al.* (2011), where the amplitude of applied deformations was negligible compared with cell size and much smaller than wall thickness. Similarly, Fernandes *et al.* (2012) measured mechanical properties on live Arabidopsis roots before and after plasmolysis and found no statistically significant effect of turgor pressure. This was attributed to the small radius of the tip used to probe cell elasticity. Our results are, however, in agreement with more recent studies in which larger indentations were employed (Beauzamy *et al.*, 2015; Weber *et al.*, 2015). It appears that in order to probe the effect of turgor pressure, one needs to apply deformations comparable with wall thickness (i.e. δ~*h*_wall_). This conclusion is based on knowledge from the mechanics of elastic shells that the component of stress in the transverse direction is changing from *–P*_T_ at the boundary with the turgid membrane to that of the atmospheric pressure in the outer layers (Landau and Lifshits, 1959). Meanwhile, the longitudinal components of the stress are only weakly changing with the position along the thickness of the wall, and hence can be safely assumed as constant. This implies that the force response towards small indentations in the transverse direction is identical irrespective of cell turgidity.

### Concluding remarks: implications of heterogeneous mechanical properties on plant cell growth

Mechanical forces in cell walls are a key feedback control mechanism in plant growth, morphogenesis, and development (Mirabet *et al.*, 2011; Peaucelle *et al.*, 2011; Kierzkowski *et al.*, 2012; Lucas *et al.*, 2013; Routier-Kierzkowska and Smith, 2013). In addition to a direct effect on signalling, mechanical stress is a part of the intercellular communication mechanism that determines a cell’s response to environmental stresses and interactions with pathogens (Santiago *et al.*, 2013). However, our understanding of the effect of mechanical forces on biological signalling is limited due to the inherent complexity and anisotropy of the plant cell wall microstructure, as well as its heterogeneous composition and distribution of polysaccharides (Cosgrove, 2000, 2005; Cosgrove and Jarvis, 2012; Park and Cosgrove, 2012; Wolf *et al.*, 2012; Ding *et al.*, 2014; Doblin *et al.*, 2014; Kafle *et al.*, 2014; Zhang *et al.*, 2014). Our results strongly suggest that variations in microstructure and composition have a major influence on mechanics, with values of Young’s modulus of the cell wall spanning several orders of magnitude. Our results contribute to the existing evidence for the heterogeneous distribution and variability of plant cell mechanical properties (Radotic *et al.*, 2012; Routier-Kierzkowska *et al.*, 2012), and for the first time provide mapping data and size characterization of such heterogeneities.

The biological implications of nano-scale mechanical heterogeneities remain unknown; we hypothesize that they may serve as an important prerequisite for development of mechanical zones and patterns which have a strong association with plant cell expansion; so-called reaction–diffusion patterns (Nagata *et al.*, 2013). In addition, the domain structure of mechanical properties may also be related to the distribution and clustering of microtubules (Xiao *et al.*, 2016). It is possible that the non-homogeneous distribution of microtubules impacts the wall mechanics via non-uniform deposition of cellulose. However, the opposite is also possible, whereby mechanical feedback favours certain clustering of cytoskeletal elements in a way analogous to how mechanical heterogeneity induces fragmentation of actin filaments in animal cells (De la Cruz *et al.*, 2015).

In conclusion, we hypothesize that multi-scale and microstructural factors may act alongside the variability in wall polymer composition, degree of cross-linking, and molecular architecture of constituent polysaccharides to affect the mechanical properties of the cell walls (Burton *et al.*, 2010; Milani *et al.*, 2011; Vogler *et al.*, 2013; Cornuault *et al.*, 2014). This work demonstrates that the presence of inherent heterogeneities in primary plant cell walls contributes to their mechanical properties. Future developments should be aimed at correlating the different inherent levels of microheterogeneities, derived from mechanical, compositional, and structural properties, into a model of primary plant cell walls that explicitly reflects its function as a ‘mechano-sensor’ regulating plant form and function.

## Supplementary data

Supplementary data are available at *JXB* online.

Figure S1. Typical examples of *Lolium* SCCs imaged in modified White’s medium under fully turgid conditions.

Figure S2. A representative TEM image of the *Lolium* SCC wall.

Figure S3. Schematic examples of force–deformation curves for different types of mechanical deformations, such as elastic, plastic, viscoelastic, and viscous.

Figure S4. Typical examples of FICs observed on *Lolium* SCCs.

Table S1. A conceptualized summary of mechanical parameters expected to be involved in mechanical responses of the cell wall during AFM nanoindentation.

Model S1. Mechanical resistor models.

Supplementary Data
