# Supplementary material for: Mapping nano-scale mechanical heterogeneity of primary plant cell walls
Source: J Exp Bot. 2016 Mar 17;67(9):2799–816. doi: 10.1093/jxb/erw117 (PMC4861025; doi:10.1093/jxb/erw117)
Supplement: Supplementary Data [file supp_67_9_2799__index.html]

Mapping nano-scale mechanical heterogeneity of primary plant cell walls — Mapping nano-scale mechanical heterogeneity of primary plant cell walls — Supplementary Data 

# Mapping nano-scale mechanical heterogeneity of primary plant cell walls

## Supplementary Data

Data files

- supplementary\_figures\_S1\_S4\_table\_S1\_\_model\_S1.pdf - Supplementary Data
