# Supplementary material for: Mapping nano-scale mechanical heterogeneity of primary plant cell walls
Source: J Exp Bot. 2016 Mar 17;67(9):2799–816. doi: 10.1093/jxb/erw117 (PMC4861025; doi:10.1093/jxb/erw117)
Supplement: Supplementary Data [file supp_erw117_supplementary_figures_S1_S4_table_S1__model_S1.pdf]

Supplementary Information

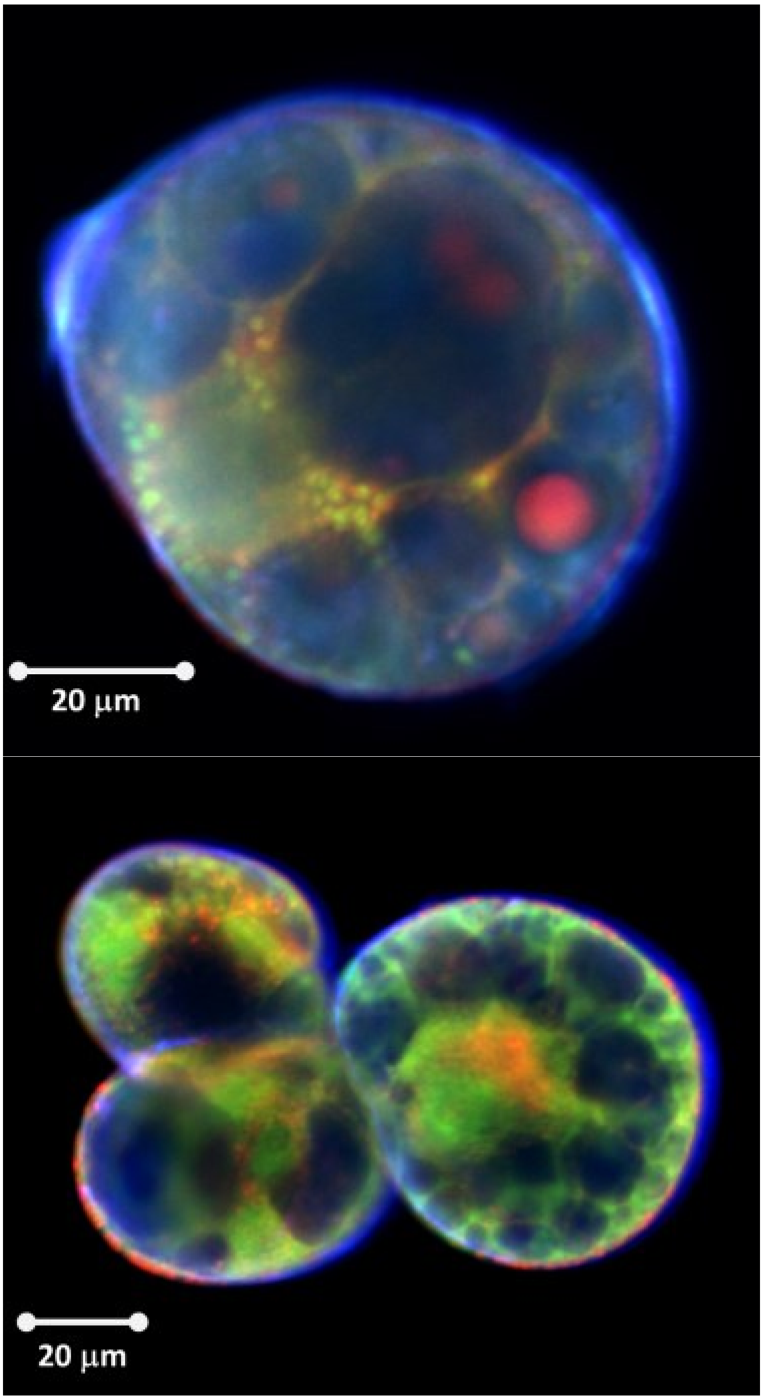

Figure S1: Typical examples of *Lolium SCCs* imaged in the modified White's medium under fully turgid conditions. The Calcofluor White staining (blue), Nile Red (red) and FDA (green) show cell wall, lipid membrane and lipid inclusions, and cell protoplast correspondingly.

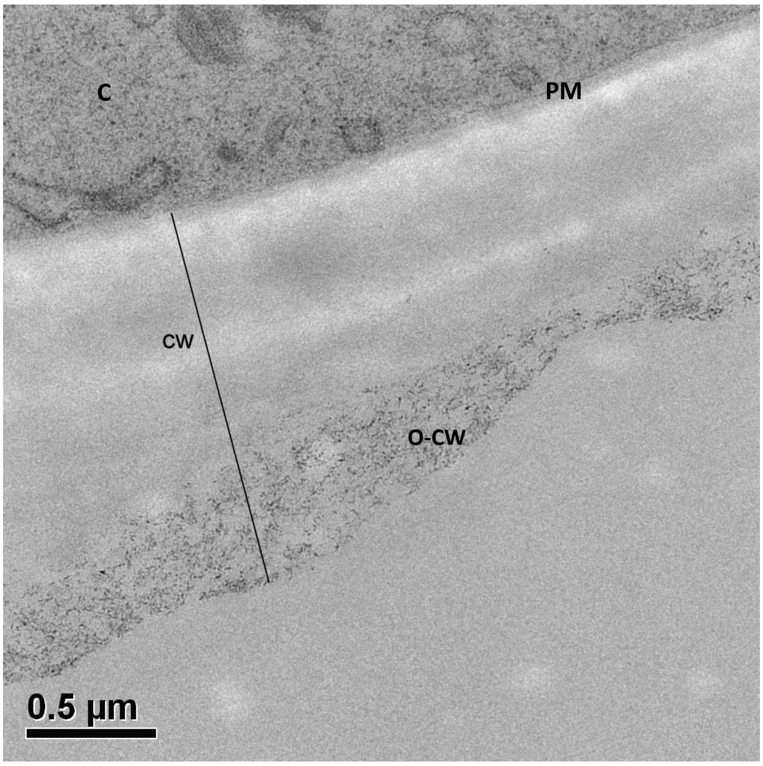

Figure S2: A representative TEM images of the *Lolium* SCC cell wall. CW – cell wall, C – cytoplasm, PM – plasma membrane. The outer wall regions (labelled ‘O-CW’) have an apparently looser structural arrangement, leading to a gel-like response on indentation as would be predicted for a loose polymer brush.

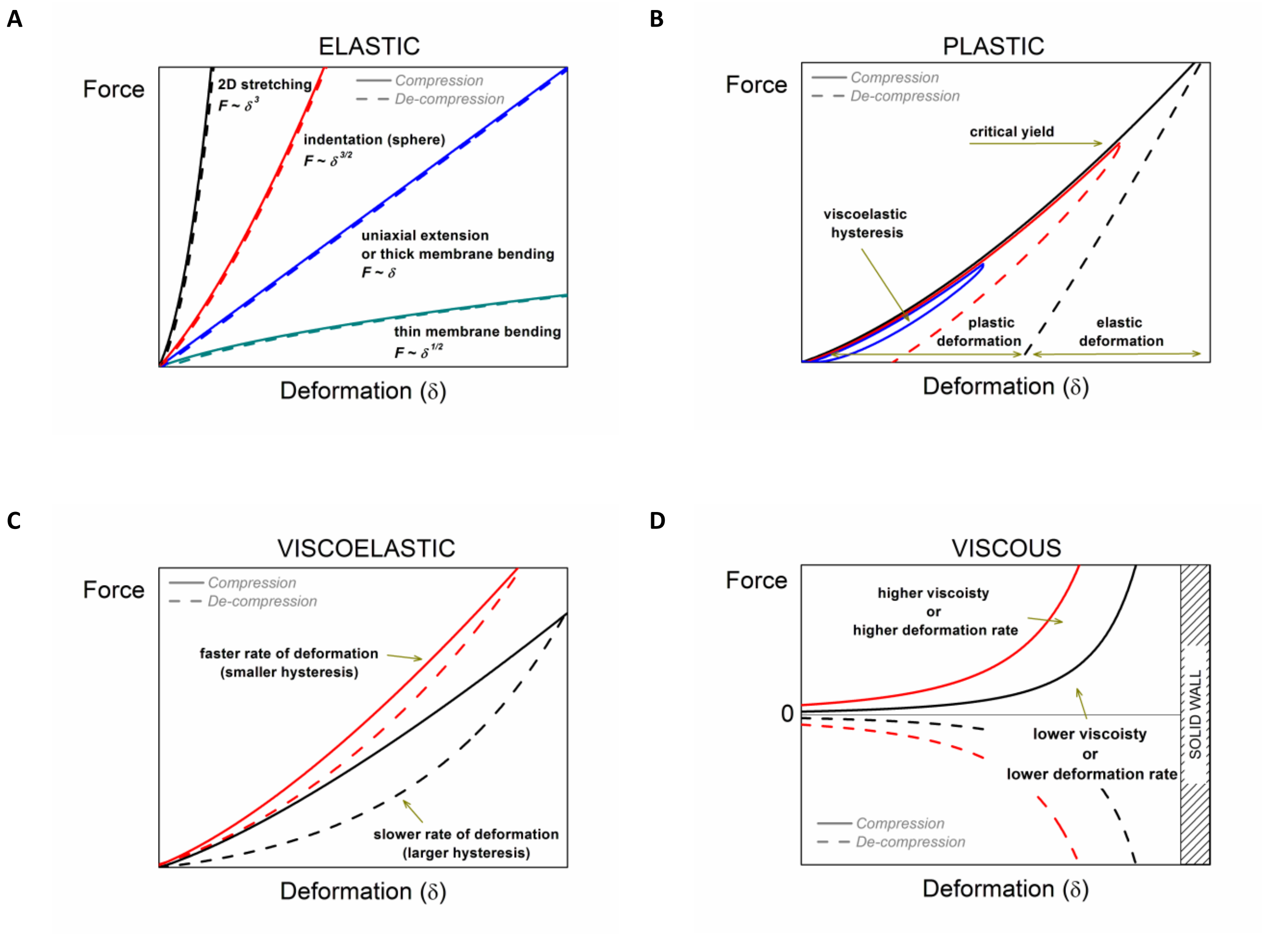

Figure S3: Schematic examples of force–deformation curves, for linear elastic (A), plastic (more generally visco-plasto-elastic) (B), visco-elastic (C), and viscous materials (D), as well as their key sensitivity variables such as deformation geometry and type of deformation, maximum compression force, deformation rate, and fluid viscosity.

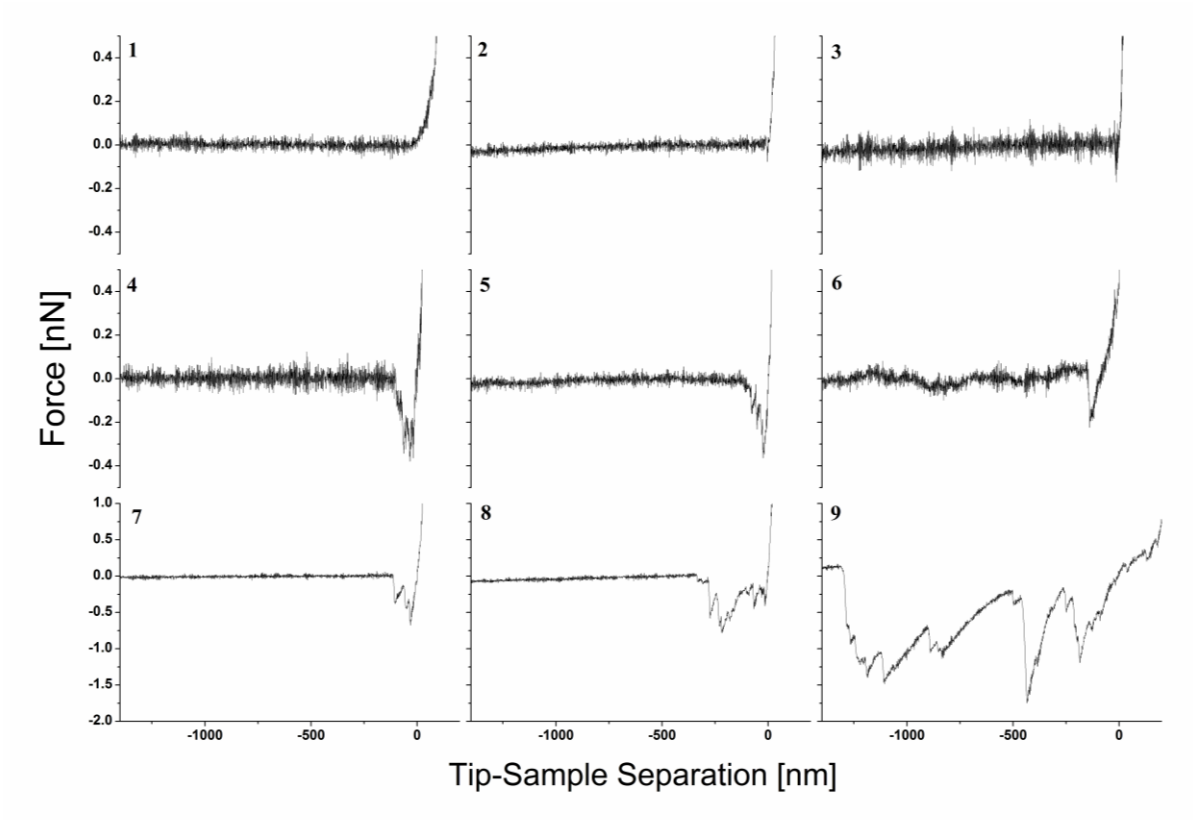

Figure S4: Typical examples of FICs observed on *Lolium* SCCs. The curve presented in Panel '1' represents ~ 75% of cases; the curves of the type presented in Panels '2' and '3' account for ~ 20% of the curves. The representative curves of the remaining 5-10% are presented in Panels '4-6'. The FIC in Panels '7', '8', and '9' are single occurrences within a set of over 350 curves.

Table S1. A conceptualised summary of mechanical parameters expected to be involved in mechanical responses of the cell wall during AFM nano-indentation.

Parameter	Dimension	Physical Meaning	Possible Structural Interpretation
$E_{\|\|}$	Pa	A measure of the stiffness of a material when deformed in a certain direction	Stretching of the polysaccharide fibril network
$E_{\perp}$	Pa		Bending of the polysaccharide fibril network, and its poroelastic compression
$\nu_{\|\|}$	dimensionles s	A measure of how a material, when compressed in one direction, expands in the other two directions	Degree of porous structure and anisotropy of the cell wall.
$\nu_{\perp}$	dimensionles s		
Y_P	Pa	A criteria of a transition beyond which a material is no longer deforms elastically (reversible)	Adhesive interaction within the polysaccharide network of the wall; loss of water during compression; defects in the cellulose micro-fibrils
τ_{VE}	S	A measure of the time scale for reorientation of the molecules in the fluid or, in the case of polymers, reflects correlated conformational transitions of polymer segments	Polymer relaxation
η	Pa·s	A measure of material resistance to gradual deformation under the applied shear or tensile stress; reflects the magnitude of internal friction between molecules	Fluid response of the outer layer of the wall. Response of the interstitial water within the wall, during poroelastic deformation

Model S1. Mechanical resistors models

In this section the models of the elastic resistors employed in this paper are described in detail.

The MRA optimisation was performed by selecting the minimum number of regimes (typically 2 or 3) from the set of equations S.1-S.7, to satisfy the goodness of fit criteria (Bonilla et al., 2015).

S1.1 Surface forces

In biological systems, surface interactions are usually the result of steric forces associated with loose polymers protruding from the surface. The exact nature of such polymeric layers is unknown, and hence its response to compression is difficult to interpret through established theories like those for well-defined grafted polymers (Milner et al., 1988). To circumvent this issue, we have opted for a simple empirical power law model:

$$F = k_p \delta^{n_p} \qquad n_p < 1 \tag{S.1}$$

A slope below unity agrees with that expected for small deformations of a brush-like layer (Subramanian et al., 1996) and experimental observation of the slow evolution of the force versus indentation curve observed in plant cells. The fitting parameters are k_p and n_p.

S1.2 Elastic shell/Circular membrane model

The indentation of an elastic shells or circular membrane displays a linear relationship between force and deformation (Begley and Mackin, 2004; Vella et al., 2012)

$$F = k_B \delta \tag{S.2}$$

The spring constant, k_B takes on two different values (corresponding to two deformation regimes) at small and large deflections compared with the shell thickness h. None of the experiments in this work involved large deflections, which means that no transitions within this regime are expected. The only fitting parameter is k_B.

S1.3 Hertzian-Sneddon (HS) deformation

Deformation of an ideal Hertzian solid by an axisymmetric punch was well-established in the pioneering work of Sneddon (Sneddon, 1965) and numerous modifications accounting for a variety of scenarios (e.g. anisotropy, multilayering) have been developed over the years. (Johnson, 1985; Popov, 2010) Here, the two basic cases of indentation with a spherical or a conical indentor are considered. Both cases, along with the sphere to cone transition, can be modelled through the following formulation developed by Briscoe et al. (1994)

$$F = 2E_{eff}\left\{a\delta - \frac{a^2}{2\tan\theta}\left[\frac{\pi}{2} - \sin^{-1}\left(\frac{b}{a}\right)\right] - \frac{a^3}{3R_{tip}} + (a^2-b^2)^{1/2}\left(\frac{b}{2\tan\theta} + \frac{a^2-b^2}{3R_{tip}}\right)\right\} \tag{S.3}$$

Where the radius of contact *a* may be derived from the following expression

$$\delta + \frac{a}{R_{tip}}((a^2-b^2)^{1/2} - a) - \frac{a}{\tan\theta}\left[\frac{\pi}{2} - \sin^{-1}\left(\frac{b}{a}\right)\right] = 0 \tag{S.4}$$

Here, E_{eff} is the effective Young's modulus, and *a*, *b* and R_{tip} are the geometric variables.

S1.4 Thin film deformation: Hertz-Chadwick model (HC)

Dimitiradis et al. (Dimitriadis et al., 2002) devised an approach for correcting Sneddon's model for samples of finite thickness indented with spherical tips. The general result is

$$F = \frac{4}{3}E_{eff}R_{tip}^{1/2}\delta^{3/2}\left\{1 - \frac{2\alpha_0}{\pi}\chi + \frac{4\alpha_0^2}{\pi^2}\chi^2 - \frac{8}{\pi^3}\left(\alpha_0^3 + \frac{4\pi^2}{15}\beta_0\right)\chi^3 + \frac{16\alpha_0}{\pi^4}\left(\alpha_0^3 + \frac{3\pi^2}{5}\beta_0\right)\chi^4\right\} \tag{S.5}$$

where $\chi = \sqrt{R_{tip}\delta}/h$ and *h* is film thickness. Parameters α_0 and β_0 depend only on the Poisson's ratio, ν. In this work the sample was assumed incompressible ($\nu = 0.5$) and bonded to the substrate, leading to $\alpha_0 = -1.7795$ and $\beta_0 = 1.0079$.

For a conical indenter, Sneddon's model has been corrected by Gavara and Chadwick (Gavara and Chadwick, 2012) to account for finite thickness

$$F = \frac{2E_{eff}\tan\theta\delta^2}{\pi}\left\{1 + 3.559\frac{\tan\theta}{\pi^2}\frac{\delta}{h} + 50.6659\tan^2\theta\left(\frac{\delta}{h}\right)^2\right\} \tag{S.6}$$

where incompressibility has again been assumed. Since both equations (S.5) and (S.6) were developed by Chadwick and co-workers, they are referred to throughout the manuscript as the Hertz-Chadwick (HC) model.

As discussed above, consideration of the transition zone between the ideally spherical indenter tip and the conical section of the indenter may be important for displacements considerably larger than R_{tip}. We have developed a computational routine to circumvent the transition between spherical and

conical indenters for the case of thin film deformation, following equations (S.5) and (S.6). The HC model has only two fitting parameters: the effective modulus E_{eff} and the film thickness, *h*.

Although this model has been developed for thin films resting on solid substrates, the mathematical description of the model can be applied to the cell wall and provide a reasonable approximation of non-linear behaviour. Firstly, the series expansion in (S.5) and (S.6) are based on the geometry of the mechanical contact. As the radius of an AFM tip is much smaller than the radius of the cell, the latter can be assumed as a flat substrate. The additional similarity is that the cell wall is resting on a turgid protoplast, whereby the stresses in the transverse direction (σ_{rr}) vary from $-P_T$ at the boundary with the plasma membrane to atmospheric pressure in the outer regions (the latter ~ 0.1 MPa and can be neglected) (Landau and Lifshits, 1959); hence for the small contact areas it is possible to assume that turgor pressure will not change upon indentation, and hence (S.5) and (S.6) can be used with reasonable caution.

S1.5 Hyperelastic deformation

The hyper-elastic model (HE) accounts for sections of the FIC in which the log-log slope is beyond that established by the thin film models (n = 5) for a displacement long enough that it cannot be attributed to distortions originating from the offset in the displacement. The HE model effectively defines the boundary of applicability of nanoindentation measurements with respect to the spring constant of the indenter probe, and it takes care of those conditions when the indentation overshoots into areas of a highly non-linear mechanical response of the material under investigation. (Chizhik et al., 1998; Tsukruk et al., 1998) The HE model simply states

$$F = A_H \delta^{n_H} \qquad n_H > 5 \qquad \text{(S.7)}$$

Therefore, it contains two fitting parameters, A_H and n_H.

References

Begley, M. R. and Mackin, T. J. (2004). Spherical indentation of freestanding circular thin films in the membrane regime. *Journal of the Mechanics and Physics of Solids* **52**, 2005-2023.

Bonilla, M. R., Stokes, J. R., Gidley, M. J. and Yakubov, G. E. (2015). Interpreting atomic force microscopy nanoindentation of hierarchical biological materials using multi-regime analysis. *Soft Matter* **11**, 1281-1292.

Briscoe, B. J., Sebastian, K. S. and Adams, M. J. (1994). The Effect of Indenter Geometry on the Elastic Response to Indentation. *Journal of Physics D-Applied Physics* **27**, 1156-1162.

Chizhik, S. A., Huang, Z., Gorbunov, V. V., Myshkin, N. K. and Tsukruk, V. V. (1998). Micromechanical properties of elastic polymeric materials as probed by scanning force microscopy. *Langmuir* **14**, 2606-2609.

Dimitriadis, E. K., Horkay, F., Maresca, J., Kachar, B. and Chadwick, R. S. (2002). Determination of elastic moduli of thin layers of soft material using the atomic force microscope. *Biophysical Journal* **82**, 2798-2810.

Gavara, N. and Chadwick, R. S. (2012). Determination of the elastic moduli of thin samples and adherent cells using conical atomic force microscope tips. *Nature Nanotechnology* **7**, 733-736.

Johnson, K. L. (1985). Contact mechanics. Cambridge, UK.: Cambridge University Press.

Landau, L. D. and Lifshits, E. M. (1959). Theory of elasticity. London; Reading, Mass.: Pergamon Press ; Addison-Wesley Pub. Co.

Milner, S. T., Witten, T. A. and Cates, M. E. (1988). Theory of The Grafted Polymer Brush. *Macromolecules* **21**, 2610-2619.

Popov, V. L. (2010). Contact Mechanics and Friction. Physical Principles and Applications. Berlin: Springer-Verlag.

Sneddon, I. N. (1965). The relation between load and penetration in the axisymmetric boussinesq problem for a punch of arbitrary profile. *International Journal of Engineering Science* **3**, 47-57.

Subramanian, G., Williams, D. R. M. and Pincus, P. A. (1996). Interaction between Finite-Sized Particles and End Grafted Polymers. *Macromolecules* **29**, 4045-4050.

Tsukruk, V. V., Huang, Z., Chizhik, S. A. and Gorbunov, V. V. (1998). Probing of micromechanical properties of compliant polymeric materials. *Journal of Materials Science* **33**, 4905-4909.

Vella, D., Ajdari, A., Vaziri, A. and Boudaoud, A. (2012). The indentation of pressurized elastic shells: from polymeric capsules to yeast cells. *Journal of the Royal Society Interface* **9**, 448-455.
